# Production and Selection of Quality Protein Popcorn Hybrids Using a Novel Ranking System and Combining Ability Estimates

**DOI:** 10.3389/fpls.2020.00698

**Published:** 2020-06-23

**Authors:** Leandra Parsons, Ying Ren, Abou Yobi, Preston Hurst, Ruthie Angelovici, Oscar Rodriguez, David R. Holding

**Affiliations:** ^1^Department of Agronomy and Horticulture, University of Nebraska – Lincoln, Lincoln, NE, United States; ^2^Center for Plant Science Innovation – Beadle Center for Biotechnology, University of Nebraska, Lincoln, NE, United States; ^3^Division of Biological Sciences and Interdisciplinary Plant Group, University of Missouri, Columbia, MO, United States; ^4^ConAgra Foods, Springfield, IN, United States

**Keywords:** hybrid-analysis, maize-breeding, popcorn, QPM, *opaque*-2

## Abstract

Popcorn varieties are agronomically sub-optimal and genetically limited compared to other maize subspecies. To increase genetic diversity and improve popcorn agronomics, dent germplasm has been introduced to popcorn with limited success and generally, major loss of popping. Between 2013 and 2018, 12 Quality Protein Popcorn (QPP) inbreds containing Quality Protein Maize (QPM) and popcorn germplasm were produced that maintained popping while carrying the *opaque-2* allele conferring elevated kernel lysine. This is an opportune trait in the growing market for healthier snacks and a model for mining QPM traits into popcorn. We crossed QPP inbreds to explore the effects of heterosis on popcorn protein, popping quality, and plant agronomics and selected hybrids for further production. To rank and intermediately prescreen hybrids, we utilized a novel hybrid-ranking model adapted from a rank summation index while examining the inbred general combining ability and hybrid specific combining ability estimates for all traits. We observed a biological manifestation of heterosis by categorizing hybrids by pedigree that resulted in a stepwise progression of trait improvement. These results corroborated our hybrid selection and offered insight in basic heterosis research. Estimates for popcorn quality and agronomic trait covariances also suggest the synergistic introgression of highly vitreous dent maize (QPM) into popcorn, providing a likely explanation for the successfully maintained vitreous endosperm, protein quality and popping traits in line with a remodeled proteome. QPP hybrids maintained improved amino acid profiles although different popping methods variably affected popcorn’s protein bound and free amino acid levels. This preliminary screening of QPP hybrids is enabling further quantitative selection for large-scale, complex trait comparison to currently marketed elite popcorn varieties.

## Introduction

**FIGURE 1:**
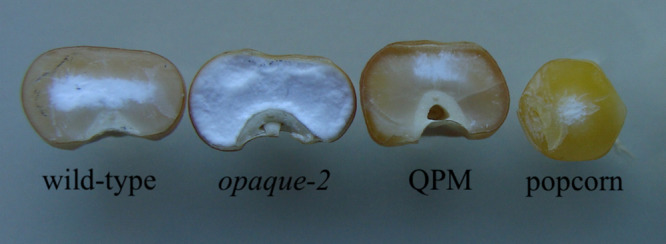
Comparative endosperm vitreousness in dent corn and popcorn backgrounds. Wild-type, *opaque-2*, and modified *opaque-2* maize kernels are from dent backgrounds. QPM has a more vitreous endosperm, like popcorn, than other dent germplasm. Popcorn has very little chalky endosperm and a round kernel morphology, determinant characteristics for popping.

Popcorn [Zea mays L. ssp everta (Sturt.) Zhuk] is a unique type of flint corn characterized by its ability to pop under heat and become an edible, direct-to-consumer snack product. Unlike dent maize, popcorn kernels are largely composed of vitreous endosperm that spans around the kernel’s small, starchy center (Figure 1). This unique morphology, coupled with appropriate moisture content, allows the kernel to expand into light flakes. The market for this popped snack-food has steadily increased for more than a decade, estimated around $9.06 billion in 2016 and projected to rise to more than $15 billion by 2023 (Dawande, 2018). Despite this persistent, growing demand, popcorn variety breeding and research has been largely overshadowed by other maize species and outpaced by its market growth (Dofing et al., 1991; Ziegler and Ashman, 1994; Kantety et al., 1995; Li et al., 2008). Due to primary selection of popping traits such as expansion volume and popability, popcorn is less optimized than other maize types in multiple agronomic traits such as pest susceptibility, stalk strength, and grain yield, and it has a relatively narrow breeding pool to integrate and improve agronomic traits (Robbins and Ashman, 1984; Sprague and Dudley, 1988; Dofing et al., 1991; Ziegler and Ashman, 1994). Previously, breeders’ attempts at introducing dent corn germplasm into popcorn to improve its agronomic fitness have met with little success because of a negative correlation between expansion volume, a key popcorn quality trait, and grain yield (Brunson, 1937; Dofing et al., 1991; Ziegler and Ashman, 1994; Pereira and Amaral Júnior, 2001; Daros et al., 2002; Li et al., 2002, 2007, 2008, 2009; Dhliwayo, 2008). However, in 2018, Ren et al. (2018) described an interpopulation breeding system between popcorn lines and dent “Quality Protein Maize” (QPM) varieties capable of increasing essential amino acid lysine in the seed proteome to more suitable levels for human dietary needs, and restored popping at early stages in the breeding program.

Dent QPM varieties were first produced by the International Maize and Wheat Improvement Center (CIMMYT) in the 1980s. Though it was known for decades prior to QPM production that the maize *opaque-2* mutation conveyed a natural biofortification of increased lysine and tryptophan in the kernel endosperm, the integration of the homozygous mutation into commercialized varieties proved challenging (Mertz et al., 1964). Due to its action as a seed storage-protein transcription factor, the knock-out of *opaque-2* manifested a soft, “opaque” endosperm phenotype (Figure 1). In their unmodified form, *Opaque-2* varieties quickly proved unfit for varietal production as they generally yielded less than its comparative germplasm and were more susceptible to fungus and pests, kernel processing damage, and lacked grower acceptance (Prasanna et al., 2001). To alleviate these setbacks, CIMMYT employed a large-scale breeding program involving multiple *opaque-2* varieties and selected moderately improved vitreousness levels through back-crossed generations. Along with the *opaque-2* mutation, CIMMYT observed the necessary introgression of unknown amino acid and endosperm vitreousness restorer genes through phenotypic selection for the biofortified, vitreous QPM end product (Babu et al., 2005; Sofi et al., 2009; Panda et al., 2010; Mbuya et al., 2011; Babu and Prasanna, 2014; Surender et al., 2014; Kostadinovic et al., 2016; Krishna et al., 2017). Though most amino acid and endosperm modifier genes remain unidentified, QTL studies have suggested that endosperm restorer genes are located on Chromosomes 1, 5, 7, and 9 (Holding et al., 2008, 2011; Babu et al., 2015). Biochemical and genetic data have suggested that increased expression and encoded protein of 27-kd γ-zein gene, in the continued presence of low α-zeins, is the most important component of modification (Geetha et al., 1991; Holding et al., 2008; Wu et al., 2010; Holding, 2014). In 2016, a 27-kd γ-zein gene duplication on Chromosome 7 was confirmed as the basis for this increase and that it is observed in all QPM varieties (Liu et al., 2016). Further investigation recently revealed this locus’s high frequency of genetic rearrangement and introduced a novel triplication allele (Liu et al., 2019). To successfully integrate the required QPM genes into popcorn backgrounds, Ren et al. (2018) utilized the visible over-production of 27-kd γ-zein along with marker-assisted selection of the *opaque-2* mutation to select for restored vitreousness of the endosperm while maintaining elevated lysine. While selecting for a QPM-like proteome, key popcorn traits such as popability, kernel morphology, and kernel size were also selected throughout the breeding program (Ren et al., 2018). Recent studies have observed that popping expansion is controlled by additive genetic factors with significant associations between multiple SNPs and elevated expansion volume (Mafra et al., 2018; Coan et al., 2019; de Lima et al., 2019; Senhorinho et al., 2019). These QTLs may be useful in future studies after associations have been validated independently, but at this stage phenotypic evaluation was determined most effective for selecting quality popcorn traits such as popability and expansion volume. After two popcorn back-crosses and multiple rounds of self-pollination, 12 BC_2_F_5_ “Quality Protein Popcorn” (QPP) lines were selected for analysis of sufficient popcorn and QPM traits. These inbred lines had highly vitreous endosperm, a QPM-like proteome, high lysine, and similar popping characteristics to the original popcorn parents (Ren et al., 2018).

The quality of popcorn endosperm protein, like normal dent maize, is low because of its deficiency in lysine and tryptophan essential amino acids (Ren et al., 2018). Previous breeding attempts have successfully introgressed the *opaque-2* allele into popcorn germplasm but have not recovered popping characteristics (Zhou et al., 2016; Adunola, 2017). These QPP inbred lines described in Ren et al. demonstrated proof-of-concept that the target traits for quality protein could be successfully integrated from QPM into popcorn without sacrificing popability (Ren et al., 2018). However, as inbreds, they were not fit for commercialized production due to inbreeding depression and unoptimized agronomic capacity. Therefore, the objectives of this study were to generate all possible QPP hybrids and select elite hybrids with superior protein quality, popcorn quality, and agronomic traits. Overall, the cumulation of these analyses enabled efficient selection of five elite QPP hybrids of three flake types out of the tested QPP hybrid population fit for future, quantitative complex trait comparison to currently marketed popcorn varieties.

## Materials and Methods

### Plant Materials and Creation of Hybrids

QPP inbred lines were produced by crossing three QPM lines, CML154Q, K0326Y, and Tx807, with four ConAgra Brands^®^ popcorn inbred lines, whose names are withheld for proprietary reasons (labeled P1–P4 to preserve identity). After F_1_ crossing in 2013, lines were back-crossed twice to the original popcorn parent and selfed five times over the course of 4 years. Phenotypically vitreous, *o2o2* homozygous BC_2_F_5_ QPP lines were produced in the winter of 2017. After evaluation, 12 BC_2_F_5_ QPP inbred lines (labeled “QPP Inbreds 1–12”) of single-seed descent from six dent × popcorn F_1_ crosses were chosen for continued analysis (Ren et al., 2018). In the summer of 2018, these lines were hand-planted and cross-pollinated in a full diallel to produce 132 QPP F_1_ hybrids. Fifteen kernels were planted per row and rows were spaced 30 inches apart. Reciprocal hybrids were designed to grow in adjacent rows for efficiency in hand-pollination and kept separate at harvest. Qualitative assessment of all maternal cobs, F_1_ grain fill, and F_1_ grain vitreousness suggested QPP inbred lines “5,” “6,” “9,” and “10” produced superior hybrids as maternal parents (Table 1). At this stage, further selection of paternal parents was not conducted to maintain a diverse array of hybrids for continued analysis. Therefore, 44 hybrids of pedigrees “5” × “1–12,” “6” × “1–12,” “9” × “1–12,” “10” × “1–12” (maternal × paternal, excluding selfing) were selected for F_1_ plant and F_2_ grain prescreening analysis in the summer of 2019. These 44 hybrids were numerically named in order of maternal parent “Inbred 5,” “Inbred 6,” “Inbred 9,” and “Inbred 10,” and paternal parent Inbred “1–12” (Table 1). After relative ranking, five QPP hybrids were chosen for final, complex trait analysis taking place in the summer of 2020.

**TABLE 1:** Depiction of inbred lines, hybrids, and pedigrees.

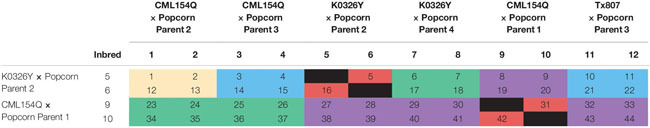

*Maternal parents shown in left two columns with pedigree history and Inbred number. Paternal parents shown horizontally in top two rows with pedigree history and Inbred number. Forty-four produced hybrids depicted as gridded squares and categorized by color according to pedigree. 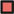 Pseudo-selfed. 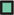 Same QPM Background. 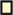 Same Popcorn Background. 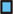 Same Popcorn Heterotic Pool. 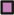 Different Popcorn Heterotic Pool.*

### 2019 Field Design

After QPP F_1_ production in 2018, 44 hybrid crosses were selected for relative intermediate analysis of F_1_ agronomic plant performance including ear size and F_2_ seed traits in the summer of 2019. Hybrids were grown in Lincoln, Nebraska and Oakley, Kansas in a Generalized Complete Block Design (GCBD) with six experimental 10-foot row units randomized per location. Original dent QPM parents, K0326Y and CML154Q, QPP Inbred 9, QPP Inbred 10, Popcorn Parent 1, and Popcorn Parent 2 were also sown and analyzed for relative comparison to hybrid progeny. Fifteen kernels were planted per row and rows were spaced 30 inches apart. Plants developed under rain fed conditions in both locations and were self-pollinated and harvested by hand. All original ConAgra popcorn inbred lines were provided by ConAgra Brands^®^. K0326Y QPM was a lab stock originally sourced from Gevers and Lake (1992), and CML154Q and Tx807 QPMs were originally obtained from the North Central Regional Plant Introduction Station as previously described (Ren et al., 2018).

### Protein Extraction and Profiling

Zein and non-zein proteins were extracted by procedures previously described (Wallace et al., 1990; Ren et al., 2018). Zein-profiles of two randomly selected F_1_ kernels from two 2018 field ears were analyzed for all 44 hybrids. Zein and non-zein profiles were analyzed on a random selection of 28 kernels from the 2019 F_2_ hybrid harvest. After selection of the five elite QPP hybrids for continued testing (Hybrids 20, 25, 28, 38, and 43), the zein profile of eight random kernels from each hybrid were analyzed to verify that the proteome was that of QPM (low α-zeins and high 27-kD γ-zein). Specifically, kernels were ground with a Wig-L-Bug^®^ dental amalgam grinder and 50 mg (±0.1 mg) of powder were used for protein extraction with a borate, β-mercaptoethanol, SDS extraction buffer. Tubes were shaken for ∼3 h at room temperature and centrifuged at full speed (13.3 g) for 10 min. Protein supernatant was further separated into zein and non-zein fractions by introducing 70% ethanol and incubating at 4°C overnight. 150 μL of both zein and non-zein fractions were placed in a vacuum desiccator centrifuge and protein precipitated. The precipitate was resuspended in 35 μL of 1X SDS-PAGE loading buffer and 5 μL samples were separated using 12% acrylamide SDS-PAGE to observe differentiable levels of staining due to particular protein abundance (termed “semi-quantitative”) for both zein and non-zein fractions.

### DNA Extraction

Leaf tissue from QPP inbreds and QPP F_1_ hybrids was collected from 2-week old seedlings and DNA was extracted according to a previously published urea-based procedure (Holding et al., 2008). DNA samples were diluted to a final concentration of ∼50 ng/μL utilizing Nanodrop^®^ and Qubit^®^ technologies.

### Genotyping the *opaque*-2 Allele

Polymerase Chain Reaction (PCR) was carried out for *opaque-2* in-gene marker umc1066 according to Ren et al. (2018). Short sequence repeat (SSR) marker umc1066 first became a useful co-dominant polymorphism for QPM conversion in 2005, and Ren et al. successfully differentiated between QPM and popcorn *opaque-2* alleles with this marker (Babu et al., 2005). Hybrid verification of *o2o2* QPM-allele homozygosity also required QPM *opaque-2* allele differentiation, which was achieved by using primers for *opaque-2* flanking marker bnlg1200, also first described by Babu et al. (2005). PCR conditions for marker bnlg1200 were to the same as marker umc1066 except annealing temperature of 55°C was used.

### Trait Analysis

Preliminary prescreening of the 44 QPP hybrids for relative competitive assessment involved measuring the following traits: germination rate (Germination), days to pollination (DAP), rot/pest susceptibility (Rot), number of ears harvested per row out of 15 seeds planted (NEH), ear length (EL), number of kernel rows per ear (RPE), ear weight (or weight of ear’s grain, WEG), 100-grain weight (100GW), kernel size (KS), kernel vitreousness (Vit), popability (PA), expansion volume (EV), flake type (FT), kernel color (KC), and amino acid profile of kernels and popped flakes in air, oil, and microwaved conditions. Germination, DAP, Rot, and NEH were measured on all plants/ears in each plot. EL, RPE, WEG, 100GW, KS, Vitreousness, PA, EV, and FT were measured on five selected ears per row and averaged for one measurement per row. EL and RPE were measured prior to shelling. WEG, KS, Vitreousness, and 100-grain weight were measured after shelling but prior to pooling the five ears’ kernels. One hundred grain weight has commonly replaced 1000-grain weight in popcorn research (Li et al., 2007, 2008; Dar et al., 2018). Final traits (PA, EV, and FT) were measured after moisture equilibration for 6 weeks in a conditioning room set at 14% moisture. Following analysis of these traits, 10 superior hybrids were selected for amino acid profiling.

Kernel Size was determined by counting the number of kernels in batches of 10-grams per ear, per row and averaging values. One-hundred grain weight was found through this estimate and appropriating the influence of each ear’s value to the final average by Ear Weight. Vitreousness was determined through light-box screening and qualitatively scored on a 1–7 scale of complete opacity to complete vitreousness, as previously described (Vivek et al., 2008; Ren et al., 2018; Supplementary Figure S1). Popability was measured by weighing one replication of 20 grams per row, counting the total number of kernels, and after popping, counting the number of unpopped kernels. Expansion volume was evaluated through popping in a domestic Orville Redenbacher Hot Air Popcorn Popper and measuring the total popped flake volume in a 1 liter cylinder. One batch of 20 g of kernels per row was measured. Flake type was determined by evaluating one randomly selected batch of 20 g of popped kernels and annotating flake type as mushroom, unilateral, bilateral, or multilateral according to previously described terminology (Eldredge and Thomas, 1959; Sweley et al., 2011).

Free and protein-bound amino acid profiles were analyzed at the University of Missouri according to published procedures (Angelovici et al., 2013; Yobi and Angelovici, 2018). Acidic hydrolysis of protein-bound amino acids destroys tryptophan and cysteine, and confounds asparagine and aspartate (Asx) and glutamate and glutamine (Glx), but all free amino acids were recovered in native form (Supplementary Tables S1, S2, S4–S9). After determining the top 10 hybrids, profiles from one replication of unpopped kernel powder per three rows per location (six samples) for each hybrid was quantified. Three kernels were ground and pooled for each replication, and all ground powder per row was used for UPLC-MSMS protein bound and free amino acid profiling. In addition to the 10 best hybrids, biological replications of QPP inbred lines (two), original proprietary popcorn (four replications for Parents 1 and 2, two replications for Parents 3 and 4), QPM dent parents (four replications for CML154Q and K0326Y, two for Tx807), and B73 (four) were also analyzed for protein-bound and free amino acid relative content. Popped flakes were also measured for free- and protein-bound amino acid determination. Four replications of five hybrids and Popcorn Parents 1 and 2 were each air-popped, microwave-popped, and oil-popped (for a total of 12 popped samples per line), and flakes were frozen in liquid nitrogen and ground in a mortar and pestle to make a fine powder.

### Statistical Analysis

#### QPP Inbred and Hybrid Analysis

The statistical model used for preliminary internal ranking of QPP hybrids is given by Equation (1):

(1) $$y_{i\,j\,k}=\mu +\beta_{i}+\tau_{j}+{\left(\beta \,\tau \right)}_{i\,j}+\epsilon_{i\,j\,k}$$

Where *y*_*ijk*_ is the hybrid’s response, *μ* is the overall mean, β_*i*_ is the environmental effect, τ_*j*_ is the treatment effect, (βτ)_*ij*_ is the location^∗^treatment interaction, and *ϵ*_*ijk*_ is the plot^∗^treatment^∗^block random effect, or error (Griffing, 1956; Addelman, 1969). The treatment effect was considered random to estimate genetic values and Type II sums of squares was used to compute the Analysis of Variance to maintain proper degrees of freedom with missing hybrid data.

Relative values of mGCA, pGCA, and SCA were measured for each trait as shown theoretically by Equations (2–5) (Griffing, 1956; Gardner, 1967):

(2) $$y_{i\,k\,l\,m}=\mu +g_{k}+g_{l}+s_{k\,l}+e_{i\,k\,l\,m}$$

(3) $$m\,G\,C\,A_{k}=\mu -y.k..$$

(4) $$pGCA_{l}=\mu -y..l.$$

(5) $$S\,C\,A_{k\,l}=y_{.kl.}.-m\,G\,C\,A_{k}-p\,G\,C\,A_{l}$$

Equation (1) was used sequentially with maternal, paternal, and hybrid treatments as random effects in ASReml-R software to estimate genetic values and standard errors (Butler, 2019). Genetic repeatability and maternal and paternal broad-sense heritabilities were calculated utilizing the genetic variance and phenotypic variance components as shown in Equation (6) (Isik et al., 2017):

(6) $$H\,y\,b\,r\,i\,d\,R\,e\,p\,e\,a\,t\,a\,b\,i\,l\,i\,t\,y\,o\,r\,I\,n\,b\,r\,e\,d\,B\,r\,o\,a\,d-S\,e\,n\,s\,e\,H\,e\,r\,i\,t\,a\,b\,i\,l\,i\,t\,y=\frac{\tau_{j}}{\sigma_{P}}$$

$$\tau_{j}=H\,y\,b\,r\,i\,d\,E\,f\,f\,e\,c\,t\,\left(S\,C\,A\right)$$

$$\tau_{j}=M\,a\,t\,e\,r\,n\,a\,l\,E\,f\,f\,e\,c\,t\,\left(m\,G\,C\,A\right)$$

$$\tau_{j}=P\,a\,t\,e\,r\,n\,a\,l\,E\,f\,f\,e\,c\,t\,\left(p\,G\,C\,A\right)$$

All analysis was conducted using R^®^ software, and the ASReml-R package was used to calculate mGCA, pGCA, SCA, co-variance, and variance of traits (Isik et al., 2017; Butler, 2019). The R-package “GGally” was used to calculate trait correlations (Schloerke et al., 2018). R-packages “lavaan,” “semPlot,” “OpenMx,” “tidyverse,” “knitr,” “kableExtra,” and “GGally” were used to conduct and visualize path analysis for comparative correlation values with EV as the main, independent variable with all variables excluding KS and DAP as dependent variables, and ear grain weight as a function of agronomic traits Germination, Rot, NEH, EL, NRE, 100GW, and Vit (Yves, 2012; Hunter, 2018; Schloerke et al., 2018; Sacha, 2019; Hao, 2019; Wickham et al., 2019; Yihui, 2020). Tukey’s Honest Significant Difference (HSD) method in R software was used to test significant differences of hybrid and parental mean trait values (R Core Team, 2018).

#### Index Selection: Adapted Rank of Sums

Selection indices are more commonly used to select inbred lines in recurrent breeding rather than ranking at the intermediate stage of hybrid selection (Hallauer and Eberhart, 1970; Johnson et al., 1988; Tardin et al., 2007; Marinho et al., 2014). This type of index requires heritability estimates coupled to repeatability to better gauge the genetic value of an inbred (Amaral Júnior et al., 2010; Lima et al., 2012; Marinho et al., 2014; De Azeredo et al., 2017; Da Luz et al., 2018). To further select the best QPP hybrids from the 44 continued crosses, a model was devised to prescreen and comparatively rank hybrids according to suggested genetic potential. The intrapopulation, relative hybrid ranking determined by the equation below reflects potential genetic value through summing the products of estimated comparative phenotype and determined economic weight of each trait. Trait estimates served as prescreening comparations capable of effective, intrapopulation ranking of the 44 QPP hybrids rather than individual quantitative values through this model. Equation (7) also includes a measure of trait repeatability in each trait’s summative ranking. For hybrid ranking, the heritability estimate was replaced with repeatability for suggested homogeneity of the hybrid, rather than heritable trait value.

(7) $$X_{h}=\sqrt{\sum_{i=1}^{m}{\left(\frac{y_{i,h}}{y_{i,m\,a\,x}}-1\right)}^{2}I_{i}(\sigma_{i,h}/\sigma_{i,\max})}$$

In the equation, *X*_*h*_ is the final, continuous rank of hybrid h′′; *y*_*i,h*_is h′′’s value of trait i′′; *y*_*i,max*_ is the superior value of trait ‘i’ across hybrids; and *I*_*i*_ is the selection intensity of trait ‘i’. Germination rate, rot susceptibility, number of ears harvested per row, ear weight, 100-grain weight, vitreousness level, popability, and expansion volume were all considered important traits in intermediate selection. Not all traits were regarded as equally important in hybrid selection, so weighting values (selection intensities) were assigned on a scale of 0–1 that graded traits based on economic importance for a commercialize line. Popability and expansion volume were assigned the heaviest weight (0.85), followed by ear weight (0.80), 100-grain weight and germination rate (0.70), vitreousness and number of ears harvested (0.60), pest/rot susceptibility and ear length (0.50), and finally number of rows per ear (0.4). Days to pollination and kernel size traits were noted for other analyses but not considered for ranking. Traits with premium values not reflected as maximum were reconfigured. For example, the rot/pest susceptibility values were subtracted from 1 (100% insusceptibility) and the differences were utilized. σ_*i,h*_ is the standard deviation of trait ‘i’ from hybrid ‘h’ and σ_*i,max*_ is the maximum standard deviation for trait i′′ across hybrids.

Final ranks were on a continuous scale with smallest values representing superior hybrids.

#### Pedigree Effect: Progression of Heterosis

The 44 QPP hybrids were separated into five categorical “hybrid” levels according to their pedigrees (Table 1). Hybrids differentiated solely by single seed descent of the same QPM and popcorn lineage were considered “pseudo-selfed.” Since inbred lines were backcrossed twice to the original popcorn parents, hybrids with the same popcorn lineage were conservatively considered 0–50% “hybrid,” while crosses with the same original QPM parent were considered closer to a true hybrid. Crosses with popcorn parents within the same heterotic group were categorized into “same heterotic group: hybrids,” and crosses between different popcorn heterotic groups were part of the “complete hybrid” group. The statistical model used for variance analysis is shown by Equation (1) inputting treatment as the “pedigree effect” on trait response. Analysis was conducted with Type II sums of squares in R^®^ software and Tukey’s HSD tests for significance (R Core Team, 2018).

## Results

### Verification of *o2o2* Genotype in QPP Hybrid F_1_ and F_2_ Kernels Through PCR and SDS-PAGE Analysis

Polymerase Chain Reaction (PCR) analysis of QPP inbred lines confirmed homozygous *opaque-2* introgression from dent parents. QPP Inbred lines 3, 9, 10, and 11 and their parental pedigrees are shown (Supplementary Figure S2). All inbreds were homozygous for the QPM *opaque-2* allele.

**FIGURE 2:**
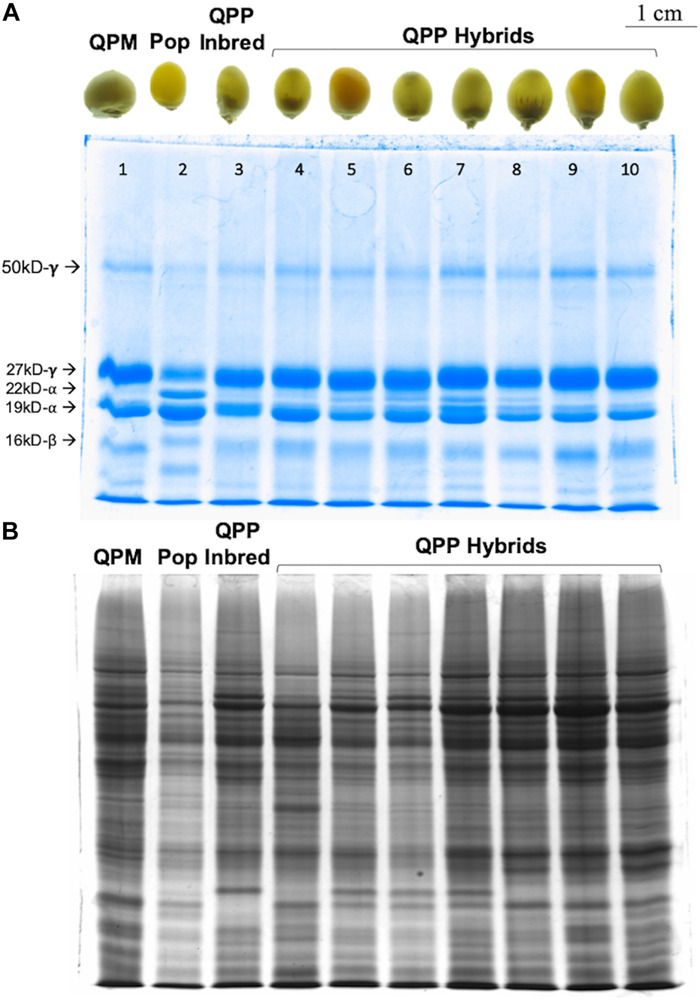
SDS-PAGE gel of random QPP hybrids verifying *o2o2* genotype. Semi-quantitative zein and non-zein extractions of random QPP hybrid kernels displayed QPM-patterned proteomes. **(A)** QPP kernels 4–10 displayed a near complete knock-down of 22kd-α zein synthesis and uniformly increased synthesis of the 27 kd-γ zein, confirming the maintenance of *o2o2* genotype from previously established inbreds. **(B)** Kernels 1 (CML154Q) and 3 (QPP Inbred 10) displayed an overall increase in non-zein production compared to Kernel 2 (Popcorn Parent 1). Random QPP hybrid kernels also displayed this trend, suggesting heightened lysine levels in the kernel due to the selected mutation. PCR verification of *o2o2* genotype in QPP inbreds is shown inSupplementary Figure S2.

SDS-PAGE analyses of F_1_ and F_2_ kernels from the 44 selected QPP hybrids confirmed the consistent QPM proteome of modified, *o2o2* mutants (Figure 2). All semi-quantitative zein SDS-PAGE analysis revealed a substantial decrease of 22-kD α-zein accumulation, varied accumulation of 19 kD α-zein, and a uniform increase in 27-kD γ-zein accumulation compared to the original popcorn, mirroring the QPM zein protein profile (Figure 2A). Moreover, F_2_ kernels showed a characteristic, although variable, relative increase in non-zein accumulation compared to the original popcorn parent indicative of increased lysine (Figure 2B). The seven random QPP hybrid kernels pictured represent the 28 kernels analyzed for zein and non-zein patterns. After ranking and selection of QPP hybrids, zein analysis of eight random kernels from elite hybrids showed the same pattern (decrease of 22-kD α-zein accumulation, varied accumulation of 19 kD α-zein, and a uniform increase in 27-kD γ-zein accumulation) (not shown). Moreover, protein-bound and free amino acid profiling of 10 select hybrids confirmed the general increase in lysine accumulation in the kernel endosperm co-validating the PCR and SDS-PAGE results of a rebalanced proteome due to introgression of the *opaque-2* recessive allele (Supplementary Tables S1, S2).

### Agronomic and Popcorn Quality Trait Evaluation of QPP Hybrids and Original Popcorn, QPM, and QPP Inbreds

**FIGURE 3:**
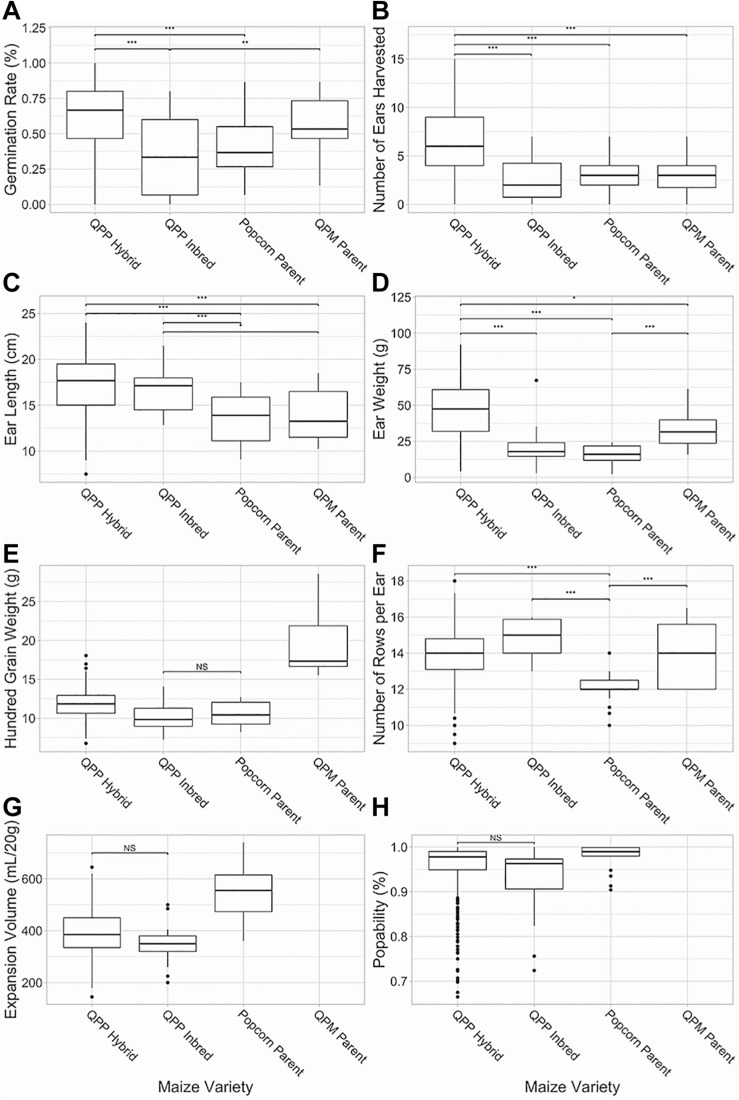
Comparison of QPP hybrids and inbreds in agronomic and popcorn quality traits. Six agronomic and two popcorn quality traits were compared between QPP hybrids and QPP, popcorn, and QPM inbreds. **(A)** Germination rate, **(B)** Number of ears harvested from single rows, **(C)** Ear lengths, **(D)** Ear weight, **(E)** Hundred grain weight, **(F)** Number of kernel rows per ear, **(G)** Expansion volume, and **(H)** Popability were compared. Popping traits were not available for QPM dent inbreds. Significant differences were noted at the *p* < 0.001, 0.001, and 0.01 levels as “***,”“**,” and “*,” respectively. “NS” denoted non-significant comparisons between groups if all other comparisons were significant. Whisker length signify range of values, boxes signify upper and lower quartiles, and the horizontal line denotes average value.

Superior agronomic performance was observed in all QPP hybrids compared to the six simultaneously grown inbred lines (*p* < 0.01; Figure 3). F_1_ hybrid plants demonstrated significantly higher germination rates and number of ears harvested from 15 planted seeds compared to QPP, Popcorn, and QPM inbreds (Figures 3A,B). Four traits out of the 12 analyzed, rot susceptibility, number of ears harvested, vitreousness, and 100-grain weight had a significant environmental interaction effects (*p* < 0.01). QPP hybrid ears were significantly longer than popcorn and QPM parents (Figure 3C). Hybrids averaged 46.6 grams per ear in grain weight, a significant improvement compared to QPP inbreds and popcorn parents (Figure 3D). Kernel sizes (as demonstrated by 100-grain weight) of all popcorn types were significantly smaller than QPM inbreds, while QPP hybrids exhibited slightly larger kernel size compared to QPP inbreds (Figure 3E). The original popcorn parents had significantly fewer number of kernel rows per ear (NRE) compared to QPM inbreds and QPP inbreds and hybrids averaged very similar NRE to QPM (Figure 3F). Flake expansion volume (EV) of QPP hybrids were on average lower than original popcorn parents (Figure 3G). QPP hybrids had a higher popability average than QPP inbreds and popability was not significantly different from the original popcorn parents (Figure 3H). These results suggest the successful selection of agronomic traits in QPP hybrids from QPM parents while sustaining popcorn quality traits from popcorn germplasm.

### Phenotypic Correlations and Path Analysis for Agronomic and Popcorn Quality Traits

**FIGURE 4:**
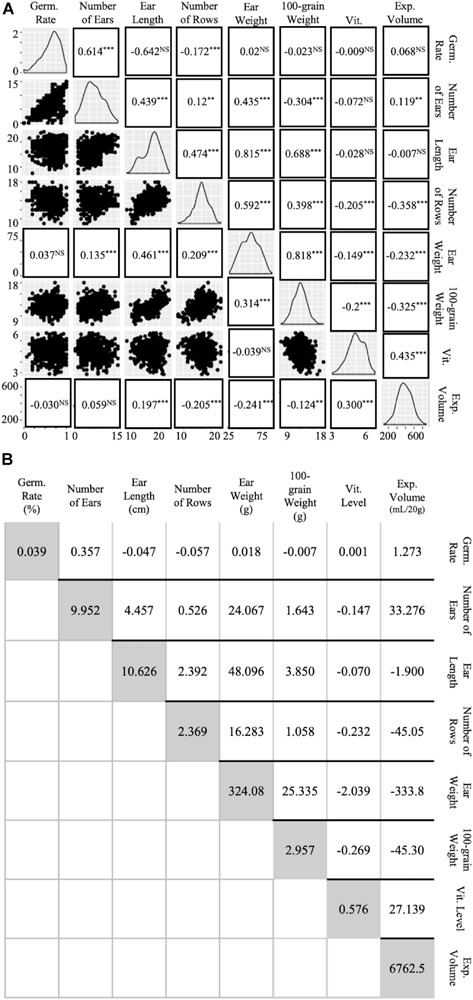
Correlations and covariances of agronomic and popping traits. High covariances and correlations were observed between multiple agronomic traits. **(A)** Agronomic and Popping Trait Correlations. Diagonal line graphs show normality of trait data. Traits correlate according to x- and y- axis labels. Dot plots under the diagonal show simple regression of traits in x-, y- columns and rows. Standardized values in replacement of dot plots under diagonal were obtained by using a path analysis. Values above the diagonal are Pearson’s Correlation Coefficients of gridded, corresponding traits. Levels of significance: *p* < 0.0001 “^∗∗∗^,” *p* < 0.001 “^∗∗^,” *p* < 0.05 “NS.” **(B)** Agronomic and Popping Trait Variances and Covariances. Covariances of traits according to row and column labeling in gridded fashion are shown above the shaded diagonal. Trait variance is described in shaded diagonal are in trait units shown on horizontal labels.

Simple regression and path analysis of preliminary trait values suggested high covariances and correlations between multiple agronomic and popcorn traits (Figure 4). Charts along the downward diagonal of Figure 4A depict the range and generally normal distribution of each of the eight traits analyzed (Figure 4A). Dot plots under the diagonal plot trait values, as described in the column and row headings, on the × and y axis for visualized regression and slope of response (Figure 4A). Values in replacement of dot plots indicate correlations derived from path analysis with EV as the independent variable and ear weight as a function of agronomic traits and vitreousness. Correlation coefficients positioned above the diagonal relate to traits as described in the column and row headings (Figure 4A) and were calculated by dividing the traits’ covariance (above darkened diagonal in Figure 4B) by both traits’ standard deviations (variances shown in diagonal, Figure 4B). Path analysis standardized coefficients and correlation coefficients complement each other in significance and trend, except for correlations between ear weight and Vit, EV and EL, and EV and number of ears harvested per row (Figure 4A). Negative coefficients were found between EV and 100-grain weight (−0.325 and −0.241), EV and ear weight (−0.232 and −0.241), and EV and number of rows per ear (−0.358 and −0.205) for phenotypic correlation and path analysis, respectively (Figure 4A). When agronomic traits were compared, high correlations between ear weight and ear length, ear weight and 100-grain weight, and 100-grain weight and ear length were calculated (Figure 4A). All three traits were evaluated to account for the possibility that kernel size and rot susceptibility could create variance in ear fill, but despite moderate occurrence of rot, strong correlations between these three traits were still observed. Additionally, though ear length variance was relatively large (10.63, Figure 4B), the trait conferred a high maternal heritability and hybrid repeatability estimate (0.432 and 0.716, respectively; Table 2 and Supplementary Table S3). Vitreousness was slightly negatively correlated to 100-grain weight, ear weight, and number of rows per ear and positively correlated to EV (0.435 and 0.300, respectively) (Figure 4A). Path analysis revealed a significant, though small, positive correlation between EV and ear length (0.197) while phenotypic correlation between vitreousness and ear length was insignificant (Figure 4A). This data supported the empirical findings that maintaining a high level of kernel vitreousness while improving popcorn agronomics, proposedly through ear length, lessened the negative side-effect on popcorn quality traits.

**TABLE 2:** Maternal and paternal general combining abilities and broad-sense heritability of all traits.

**Inbred**		**Germination rate (%)**	**Days to pollinating**	**Rot susceptibility (%)**	**Number of ears harvested per rows**	**Ear length (cm)**	**Number of rows per ear**	**Ear weight (g)**	**Kernel size (#/10 g)**	**Vitreousness level**	**Hundred grain weight exp. volume (g) (mL/20 g)**		**Pop-ability (%)**
mGCA	5	0.012	0.915	0.020	–0.653	–1.419	–0.680	–9.033	7.407	0.110	–1.030	25.788	0.002
	6	0.114	0.647	0.064	0.635	–1.715	–0.897	–10.186	6.089	0.055	–0.764	30.836	0.006
	9	–0.038	–0.708	–0.075	0.374	1.950	0.465	11.841	–7.732	–0.048	1.084	–35.859	–0.010
	10	–0.088	–0.854	–0.010	–0.356	1.184	1.112	7.378	–5.764	–0.118	0.709	–20.765	0.003
Standard error		0.006	0.753	0.004	0.393	2.82	0.758	105.377	51.430	0.015	0.911	966.98	0.000
Heritability		0.163	0.123	0.059	0.049	0.432	0.358	0.448	0.368	0.024	0.415	0.173	0.026
pGCA	1	0.048	0.220	0.000	0.519	–1.283	–1.235	–3.867	–0.135	–0.148	0.051	18.245	–0.009
	2	–0.122	1.554	0.000	–1.293	–0.334	–0.776	–5.470	2.646	–0.315	–0.258	4.426	–0.008
	3	0.013	–0.914	0.000	0.208	–0.215	0.658	2.234	–1.423	–0.682	0.074	–25.857	0.001
	4	0.010	0.736	0.000	0.070	–1.066	0.837	1.537	0.669	–0.281	–0.134	–28.950	–0.005
	5	–0.006	–0.139	0.000	–0.150	–0.023	–0.982	–4.466	5.214	0.798	–0.519	53.808	0.014
	6	–0.050	0.300	0.000	–0.499	–0.042	–0.888	–4.474	4.136	0.698	–0.500	72.878	0.018
	7	–0.131	0.674	0.000	–1.689	0.401	0.267	3.179	–6.910	–0.404	0.989	–26.492	0.004
	8	0.048	1.105	0.000	0.346	–0.611	–0.307	–4.625	5.485	–0.266	–0.765	25.927	0.003
	9	0.039	–1.315	0.000	0.697	1.330	0.561	4.611	–2.976	0.578	0.297	9.362	0.009
	10	0.023	–1.126	0.000	0.306	1.670	0.523	2.197	–0.583	0.494	0.027	33.080	0.020
	11	0.075	–0.688	0.000	0.795	–0.343	0.920	3.512	–1.237	–0.531	0.067	–57.287	–0.009
	12	0.054	–0.407	0.000	0.691	0.515	0.422	5.632	4.887	0.059	0.671	–79.139	–0.039
Standard error		0.003	0.473	0.00	0.401	0.447	0.298	11.77	9.07	0.119	0.146	947.81	0.0001
Heritability		0.117	0.138	0.000	0.092	0.124	0.274	0.086	0.115	0.445	0.119	0.322	0.123

*mGCA and pGCA values for all traits are listed as columns with broad-sense heritability estimates shown in gray. All combining ability estimates are in units according to trait calculated.*

### Pedigree Analysis of QPP Hybrids

**FIGURE 5:**
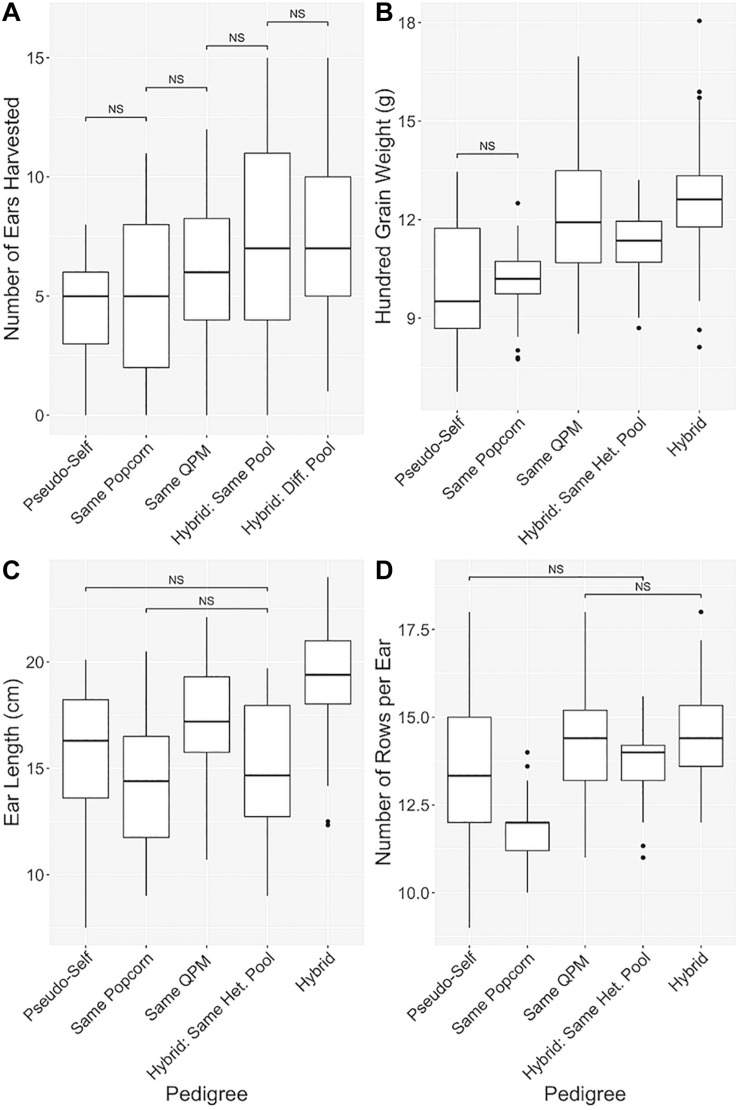
Manifestation of hybrid vigor through pedigree analysis. Pedigree-based categorical grouping of hybrids for agronomic comparison. In order of increasing genetic diversity, hybrids were sorted into “Pseudo-self,” “Same Popcorn,” “Same QPM,” “Hybrid: Same Het. Pool,” and “Hybrid” categories. Traits analyzed were **(A)** Number of ears harvested per row, **(B)** 100-grain weight (g), **(C)** Ear length (cm), and **(D)** Number of rows per ear. “NS” denoted non-significant comparisons between groups with all other comparisons as significant. Whisker length signify range of values, boxes signify upper and lower quartiles, and the horizontal line denotes average value.

Hybrids were categorically separated into five groups in order of increasing genetic diversity (Ren et al., 2018; Table 1 and Figure 5). All agronomic traits exhibited a similar trend of improvement from the pseudo-selfed lines to the complete-hybrid groups. “Ears harvested per row” averages between categorical groups slowly inclined, and significant differences were found between all categories one step apart (Figure 5A). One hundred grain weight values exhibited a similar trend, except hybrids within the same QPM background had a slightly larger average than hybrids in the same heterotic group (Figure 5B). QPP hybrids from different heterotic groups averaged the highest ear length while categories involving the same popcorn background or heterotic pool notably decreased compared to the same QPM or different heterotic pool categories (Figure 5C). A dragging trend in similar popcorn genetics (backgrounds and heterotic pools) was also noticed in NRE (Figure 5D). Like EL, groups with the same popcorn background were significantly stunted in kernel row number, averaging almost the same as popcorn parental inbreds (11.78 ± 0.809 and 12.11 ± 0.928, respectively).

Principle Component Analysis of all trait data supported the validity of these categories and subsequent heterotic trend. A composite 96.56% of data variance was explained by the first two principle components (Supplementary Figure S3). QPM parents K0326Y and CML154Q fell far from all other popcorn related lines and were clustered into the same group as other inbreds. All “Same Popcorn Background” hybrids fell in/near the inbred cluster (Supplementary Figure S3). These components were determined predominantly by variances associated with a kernel size, ear weight, and maturity (Supplementary Figure S3). Hybrids of the same heterotic group displayed a tight cluster separated completely from hybrids of different heterotic groups, though both overlapped with “Pseudo-self” and “Same QPM Background” clusters (Supplementary Figure S3). Complete hybrids notably separated themselves from hybrids from the same heterotic pool due to heavier ear weight and longer ear length, while hybrids from the same heterotic group favored smaller, more popcorn-like kernel sizes and later maturity (Supplementary Figure S3). Like Figure 5, progression in agronomic improvement, specifically in ear length, ear weight, and kernel size, was evident through PCA of the five genetically distinct categories of QPP hybrids (Figure 5 and Supplementary Figure S3).

### QPP Hybrid and Inbred Flake Type Analysis

**FIGURE 6:**
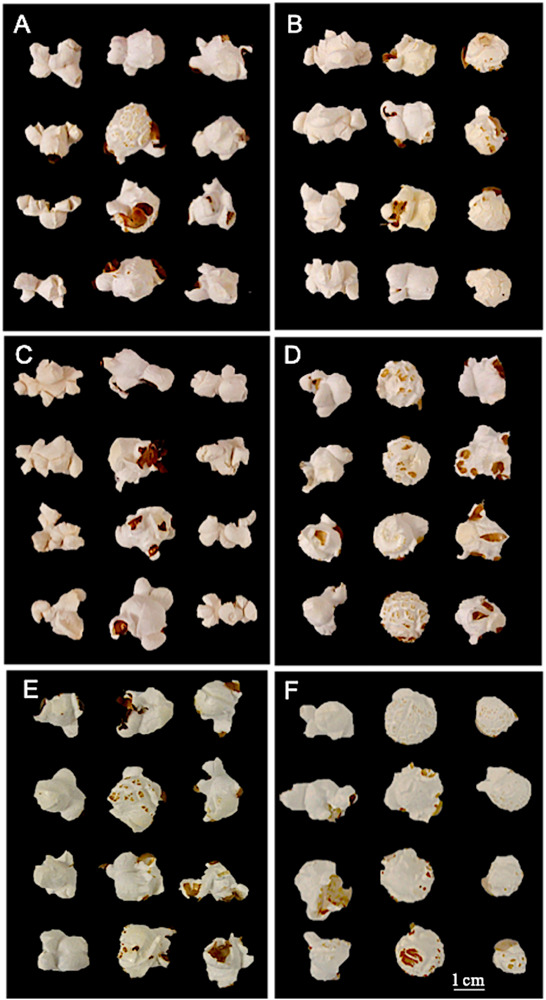
Inbred and hybrid flake morphology. **(A)** First column: maternal parent 6 (bilateral morphology); Second column: Hybrid 20; Third column: paternal parent 10. **(B)** First column: maternal parent 9; Second column: Hybrid 25 (unilateral morphology); Third column: paternal parent 3. **(C)** First column: maternal parent 9; Second column: Hybrid 28 (multilateral morphology); Third column: paternal parent 6. **(D)** First column: maternal parent 10; Second column: Hybrid 34 (mushroom morphology); Third column: paternal parent 1. **(E)** First column: maternal parent 10; Second column: Hybrid 38; Third column: paternal parent 5. **(F)** First column: maternal parent 10; Second column: Hybrid 43; Third column: paternal parent 11.

Utilizing unilateral, bilateral, multilateral, and mushroom terminology (Sweley et al., 2011), all QPP inbreds and hybrids were categorized into one or two flake types (Table 3 and Figure 6). Bilateral flake types were not observed across all hybrids (Table 3). Hybrids from maternal parents 5, 6, and 10 seemed to display either unilateral or mushroom flakes, in agreement with inbred morphology, while hybrids from maternal parent 6 had a more diverse morphology of mushroom or multilateral flakes (Figure 6 and Table 3). Paternal parents 11 and 12 also exhibited a mushroom flake in all progeny with different degrees of uniformity, reflecting the flake type of the inbreds (Figure 6 and Table 3). Hybrids involving Inbreds 3 and 4 also popped with mushroom flakes like the inbreds, though notably crosses 25 and 26 had uniform unilateral flakes, like Inbred 9. Out of the 22 crosses involving maternal lines 9 and 10, nearly half displayed uniformly unilateral flakes (Table 3). In contrast, all hybrids from maternal Inbred 6 had mixed morphologies except for hybrid 19, which was multilateral (Table 3). Nine hybrids in all displayed some occurrence of multilateral flakes and the morphology was tested for association with high EV, but no correlation was found. Hybrids 23–26 exhibited uniformly unilateral flakes compared to Hybrids 34–37 that displayed near uniform mushroom morphology (Table 3). Half of hybrids from Inbreds 1 and 2 exhibited mushroom morphology though these inbreds had a multilateral morphology (Figure 6). Inbreds 11 and 12 exhibited the mushroom morphology successfully in almost all hybrids, including those with Inbred 9 as the maternal parent (Table 3). Before hybrid ranking and selection, it was determined that diversity in flake type would be maintained in the final list of chosen hybrids. Thus, after ranking and inbred analysis, final hybrids with two uniformly unilateral, two unilateral and multilateral mixed, and one mushroom morphology were chosen for continued analysis.

**TABLE 3:** Flake morphologies in hybrid popped flakes.

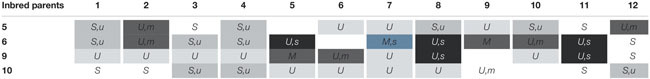

*One sample of 20 grams of popped kernels were examined and flake types assigned for each hybrid. “S” is Mushroom morphology. “U” is Unilateral morphology. “M” is multilateral morphology. Capital lettering suggests the prevailing flake type, while lower-case suggests a secondary flake type, if applicable.*

### Novel Hybrid Ranking System Identified Top QPP Hybrids

**FIGURE 7:**
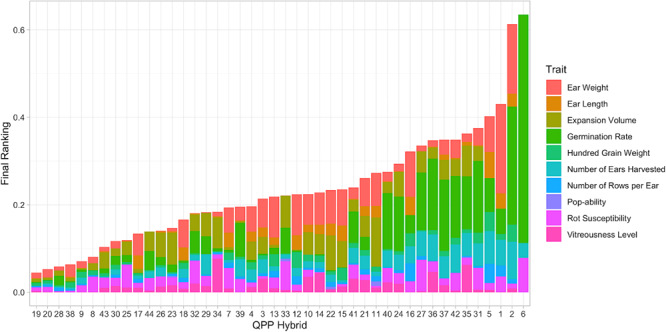
Categorized results from hybrid ranking model. Elite hybrids determined from the Ranking Model are listed from left to right as summed ranking value increases. Lower score indicates less distance from maximum trait value, i.e., Hybrid 19 ranked best compared to all hybrids. Stacked bars represent individual trait influence on each hybrid’s overall rank.

All relevant trait data was imputed into the ranking model as shown by Equation (7). After computation, each hybrid was assigned a final ranking number that was the composite of 10 trait values (Figure 7). Hybrid 6 held the highest value (signifying the *worst* ranking of all hybrids), which was mostly due to its relatively poor germination (Figure 7). Hybrids 19, 20, 28, 38, 9, 8, 43, 30, 25, and 17 were identified as the top 10 (Figure 7). Hybrids 19 and 20 ranked highest with minimal deviations from the maximum trait values in all traits. Hybrid 20 was slightly hindered by its lower EV, as was Hybrid 28’s lower 100-grain weight. Hybrids 8, 25, and 32 had large rot values but they did not affect ear weight (Figure 7). Hybrids 30 and 25 were very similar in rank since Hybrid 30 had a more inferior ear weight with minimal rot susceptibility. Hybrid 43, 44, 26, and 23 were hindered by expansion volume, which was more noteworthy for Hybrids 23 and 26 since they expanded unilaterally compared to Hybrids 43 and 44 which expanded in mushroom morphology (Table 3). Hybrid 17 ranked tenth, with a value predominantly composed of ear weight and ear length marks (Figure 7).

The summation of all preliminary evaluations enabled the holistic ranking of hybrids by overall genetic value, analyses akin to other selection indices. However, maintaining individual trait distinctions and extent of effect enabled a thorough understanding of hybrid rank. The top nine hybrids: 19, 20, 28, 38, 9, 8, 43, 30, 25, and hybrid 23 (lower due to EV) were chosen for amino acid profiling and further selection.

### Assessment of Top Hybrids Utilizing General and Specific Combining Ability Estimates

Hybrid analysis enabled maternal and paternal GCA values to be assigned according to offspring productivity. Maternal GCA values were only assigned for Inbreds 5, 6, 9, and 10, and paternal values were calculated for all QPP inbreds (Table 2). Due to inbred similarity in original pedigree (shown in Table 1), most combining ability values were similar for pairs of inbreds with the same QPM and popcorn parents. Trends were observed between the maternal pairs of Inbreds 5 and 6 and Inbreds 9 and 10. Ear weight maternal and paternal combining abilities were not used in downstream analysis due to large standard error and insignificant differences. mGCA estimates for Inbreds 9 and 10 (CML154Q × Popcorn Parent 1) were significantly higher than Inbreds 5 and 6 in agronomic traits ear length, number of rows per ear, and 100-grain weight (Table 2). These traits also had the highest maternal heritability values at 0.432, and 0.415 for EL, and 100-grain weight, respectively. Higher heritable values coupled to significant differences in maternal general combining ability values suggested that Inbreds 9 and 10 were superior maternal parents agronomically. Inbreds 5 and 6 held the highest expansion volume GCAs for all parents, though these values were considered insignificant. However, the trend in higher EV GCA values for these inbreds suggested that Inbreds 5 and 6 were strong paternal parents in popcorn quality traits, especially when considering they also held the highest popability pGCAs and paternal heritabilities were larger than maternal for both EV and popability, at 0.322 and 0.123, respectively (Table 2). Moreover, the heritability estimates for vitreousness varied substantially between maternal and paternal parents; with values of 0.024 and 0.445, respectively. Therefore, Inbreds 5 and 6 again stood out as premier paternal parents with significantly highest vitreousness pGCA values (Table 2). The combination of Inbreds 9 and 10 as maternal parents and Inbreds 5 and 6 as paternal parents suggested premier crosses, aiding the eventual selection of both Hybrids 28 and 38 rather than their reciprocals Hybrids 19 and 9 (Table 2). Hybrid 20 was favored over Hybrid 19 due to Inbred 10’s larger popcorn quality trait pGCA value for Popability, which is highly correlated to EV, compared to Inbred 9 (Table 2).

Specific Combining Ability values, standard error, and genetic repeatability estimates were calculated for all QPP hybrids (Supplementary Table S3). High standard errors for EV and ear weight in both general and specific combining ability estimates limited their direct use for QPP hybrid selection; however, calculated significant correlations between traits such as ear length and ear weight, and popability and EV, enabled discriminatory selection of elite hybrids utilizing more accurate inbred genetic values coupled to heritability and repeatability estimates. The ranking system allowed for a direct, preliminary narrowing of best hybrids for further testing, after which heritability and repeatability estimates with standard error determined the reliability of combining ability values that guided final selection. Due to high heritability and low standard error, ear length and Vitreousness SCA values became the premier traits for final selection. Hybrids 20, 25, 28, 38, and 43 all exhibited positive EL SCAs and Hybrids 20, 28, 38, and 43 held positive Vitreousness SCAs.

### Highly Ranked QPP Hybrids Showed Elevated Lysine in Raw and Popped Kernel Flours

**FIGURE 8:**
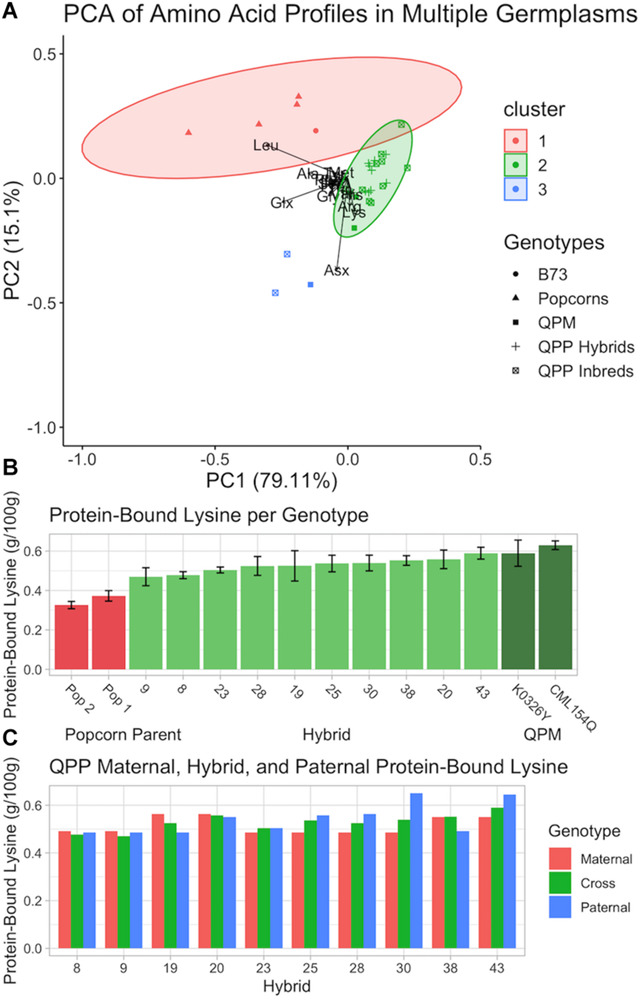
Analysis of protein-bound amino acid composition in various genotypes in flour from raw kernels. **(A)** Principle Component Analysis of protein-bound amino acids in ground powder of B73, QPP Inbreds, QPP Hybrids, Popcorn, and QPM germplasms. Various shapes represent different germplasms. **(B)** Protein-bound lysine (g/100 g) of two popcorn parents, two QPM parents, and 10 QPP hybrids with standard deviation error bars. **(C)** Protein-bound lysine (g/100 g) of QPP hybrids and respective maternal and paternal parents. Standard errors are not shown and available in Supplementary Table S1.

After the 10 best hybrids were selected, flour from raw kernels and air, microwave, and oil popped flakes were analyzed for protein-bound and free amino acids. Principle Component Analysis of protein-bound raw kernel amino acid profiles suggested a major shift in the QPP proteome away from popcorn parents and toward QPM (Figure 8A). Genotypes were grouped into two main clusters. Cluster one was composed of popcorn parents (and B73 dent corn) and cluster two of QPP and QPM germplasm with the overlap of one genotype (QPP Inbred 9) (Figure 8A). CML154Q and K0326Y were grouped into cluster two and indistinguishable from QPP inbreds and hybrids (Figure 8A). QPP Inbreds 7 and 8 and QPM line Tx807 displayed a distinctive protein-bound amino acid profile compared to all other lines and formed cluster three, though too few points were available to calculate an ellipse (Figure 8A and Supplementary Table S1). With histidine, methionine, and lysine as the exceptions, Inbreds 7 and 8 consistently had the highest protein-bound amino acid levels, though this trend did not hold with free amino acid values (Supplementary Tables S1, S2). Principle Component Analysis of free raw kernel amino acids instead suggested a general distinction between QPP inbreds and QPP hybrids (Supplementary Figure S4). Like the protein-bound analysis, Inbred 9 bordered the popcorn parent cluster, and K0326Y, Tx807, and QPP Inbreds 10, 8, and 6 overlapped with QPP hybrids (Supplementary Figure S4). All other QPP Inbreds and CML154Q formed a separate group with characteristically high levels of proline, aspartate, glutamine, glutamine, and alanine (Supplementary Figure S4).

To further confirm the homozygous introgression of the QPM *opaque-2* allele, free and protein-bound lysine levels in raw kernels were specifically compared between QPP hybrids and original QPM and popcorn parents (Figure 8B). Significant increases in QPP lysine levels compared to the original popcorn parents were observed in all hybrids (Figure 8B). K0326Y and CML154Q maintained slightly higher lysine levels than QPP hybrids, though not always significant (Figure 8B). QPP Hybrids 43, 20, and 38 had the highest protein-bound lysine levels (0.589, 0.558, and 0.552 g/100 g, respectively) compared to CML154Q and K0326Y (0.629 and 0.589 g/100 g, respectively) (Figure 8B and Supplementary Table S1). Overall, the 10 tested QPP hybrids had 1.45- and 3.86-fold increases in raw kernel, protein-bound and free lysine content over popcorn parents, respectively (Supplementary Tables S1, S2). Specifically, the five selected hybrids for further analysis (Hybrids 20, 25, 28, 38, and 43) held 1.52- and 4.45-fold increases in protein-bound and free, raw kernel lysine levels, verifying the biofortification of the popcorn proteome to pattern that of QPM.

As pedigree analysis of agronomic traits revealed a manifestation of heterosis due to genetic diversity, raw kernel protein-bound lysine levels were compared between QPP hybrids and their inbred parents (Figure 8C). An additive effect was observed in all cases except Hybrid 38 (Figure 8C). Hybrid 38 and Inbred 10’s lysine levels were significantly larger than Inbred 5, suggesting a dominant heterotic effect in this singular case (Supplementary Table S1). However, with 9 out of 10 parental pairs holding an additive effect, the trend suggests that lysine level in QPP crosses can be moderately predicted. Similar comparative analysis between parents and crosses were conducted on all protein-bound amino acids, and over-dominant trends, or the synergistic effect of a heterozygous state of alleles to confer a superior phenotype, in this case elevated amino acid abundance, in the hybrid compared to the parental inbreds, were noted for alanine, arginine, aspartate/asparagine, histidine, leucine, and methionine (Shapira and David, 2016). Additive and/or dominant trends were suggested in glutamate/glutamine, glycine, phenylalanine, serine, and isoleucine, and exclusively additive trends were identified in proline, threonine, and tyrosine (Supplementary Table S1). Though verifying effects would require additional testing, consistent trends in particular amino acids suggest moderate predictability of hybrid amino acid levels according to inbred values and could guide selective breeding accordingly.

**FIGURE 9:**
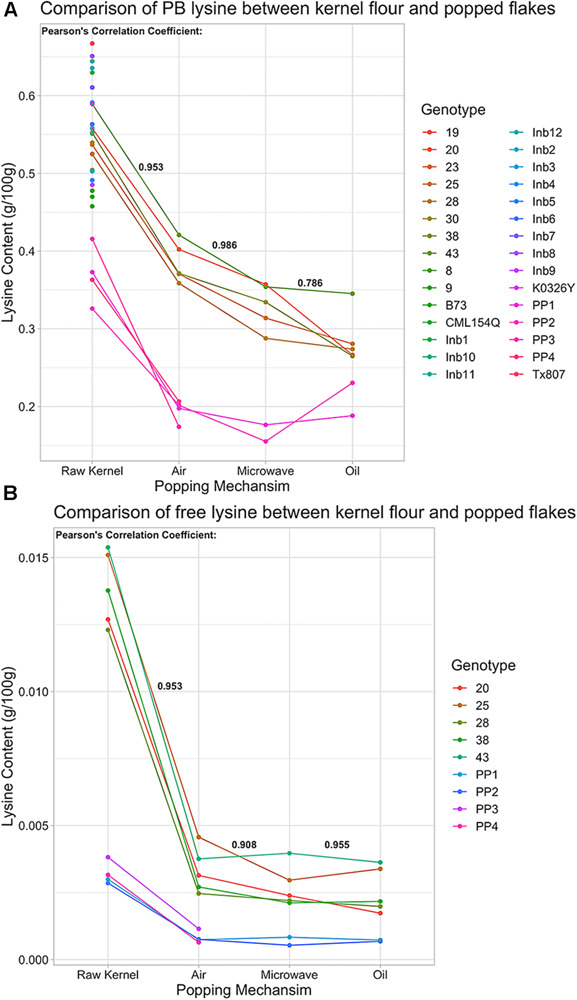
Protein-bound and free lysine content of QPP Hybrids, Inbreds, QPM, and Popcorn Germplasm in raw kernel and popped flakes. **(A)** Protein-bound lysine content (g/100 g) in various germplasm samples under air, microwave, or oil popping conditions compared to raw kernel powder. Points along vertical “Raw Kernel” axis are lysine levels from germplasm that was not popped. **(B)** Free lysine (g/100 g) in multiple germplasm samples under air, microwave, or oil popping conditions compared to raw kernel powder. Correlation Coefficients between protein-bound and free lysine levels in raw kernel and air popped flakes, air popped flakes and microwaved flakes, and microwaved flakes and oil popped flakes were calculated and are in respective positions in bold. Genotypes with solely a numbered label signify QPP hybrids, QPP Inbreds are named “Inb” preceding inbred number, and “PP1” – “PP4” represent “Popcorn Parent 1–4,” respectively.

The five chosen QPP hybrids and two popcorn parents were popped using air, oil, and microwave methods to identify correlations in amino acid changes between ground powder and several different popping methods. QPP hybrids maintained higher lysine levels than popcorn parents across all popping methods, though protein-bound and free lysine levels decreased to different extents when kernels were popped (Figure 9). Air popping appeared to result in the least loss of protein-bound lysine, decreasing contents on average by ∼0.15 g/100 g lysine (Figure 9A and Supplementary Tables S1, S4). Values suggested that microwave and oil popping decreased protein-bound lysine content more than air popping, though confidence intervals overlap (Figure 9A and Supplementary Tables S4–S6).

To ascertain the consistency in lysine loss due to popping methods, correlation coefficients were calculated between all four treatments – raw powder and microwave, oil, and air popping, and a highly correlative trend in lysine loss was observed (*p* < 0.05; Figure 9A). With such a consistent decrease in protein-bound lysine due to popping, all other amino acids were examined for uniformity and extent of decline. Most protein-bound amino acid levels correlated with a coefficient higher than 0.700 between ground powder, air, microwave, and popped methods. Proline, threonine, and asparagine/aspartate’s oil method correlations, isoleucine and serine’s oil method correlations to air and microwave popping, and almost all correlations in glycine and valine levels were low. The amount of change varied by amino acid, commonly increasing in abundance after popping by air and microwave methods (ex. glycine, isoleucine, and leucine; Supplementary Tables S1, S4–S6). Though levels changed by varying percentages depending on amino acid and method, high correlations between raw kernel and air and microwave popped flake protein-bound amino acid values suggest a consistent effect of popping on protein-bound amino acid level variations (Supplementary Tables S1, S4–S6). Like lysine levels, most QPP protein-bound amino acids supported a similar trend of insignificantly different amounts in air and microwave popping methods and slightly lower abundances with varying levels of significance in oil-popped flakes (Supplementary Tables S1, S5–S7). Though confidence intervals were wide across popping methods and genotypes, comparative analysis between QPP hybrids and popcorn parents suggested that popcorn germplasm held higher protein-bound serine, phenylalanine, methionine, alanine, tyrosine, isoleucine, leucine, and glutamate/glutamine levels than QPP, while QPP hybrids exhibited higher levels of histidine, arginine, asparagine/aspartate, and lysine levels than popcorn parents (Supplementary Table S1). Ground samples of QPP hybrids that were not tested in the popped state also exhibited superior lysine levels compared to popcorn parents, and high correlations between raw kernel and popping methods suggest that all hybrids are superior in lysine levels regardless of popping method employed, a trend further exemplified in free amino acid levels (Figures 9A,B and Supplementary Figure S5A).

Free amino acid analysis revealed that QPP hybrids had a higher abundance of free amino acids in all residues except serine and methionine compared to popcorn parents (Supplementary Tables S2, S7–S9). Like protein-bound values, free amino acid levels suggested similar trends in declined abundance after all popping methods, with cysteine and threonine values as exceptions (Supplementary Figure S5A and Supplementary Tables S2, S7–S9). Like protein-bound residues, high correlations (>0.7) were observed between almost all popping methods and raw powder in free amino acid comparisons, offering further confidence that popping has a reliable, consistent effect on the proteome and amino acid fluctuations. Unlike protein-bound values, free amino acids suggested a uniform trend in decreased residue abundance due to all popping methods (except threonine and cysteine; Figure 9B and Supplementary Figure S5). On average, QPP hybrids sustained a 0.0087 g/100 g loss of free lysine and popcorn germplasm sustained a 0.0023 g/100 g loss when air popped, 72.3 and 74%, respectively, of the raw kernel free lysine level (Figure 9B and Supplementary Tables S1, S2, S4, S7). Since QPM conveys the characteristic increase of essential amino acids lysine and tryptophan, free tryptophan levels of QPP hybrids were examined and held significantly superior levels compared to popcorn parents and, like protein-bound lysine, most hybrids held insignificantly different levels of free tryptophan compared to QPM (Supplementary Figure S5B).

## Discussion

### The Popcorn Market: Future Prospects

U.S. consumer trends veering toward a more health-consciousness and continually fast-paced lifestyle have correlatively increased with the popcorn market, which is expected to grow at an annual rate of 7.6% over the next 3 years (Dawande, 2018). Popcorn producers have responded with more detailed labeling describing caloric intake, offering all-natural, clean label options, and introducing more flavor options to the consumer (Mordor Intelligence, 2018). Successful dent by popcorn crosses have resulted in improved agronomics with enhanced flavor profiles of the popped flakes; however, maintaining popability and expansion volume remains a key challenge (Crumbaker et al., 1949; Johnson and Eldredge, 1953; Robbins and Ashman, 1984). In this study, the use of Quality Protein Maize varieties in QPM by popcorn crosses had a triplicate effect of improving popcorn agronomics, seed protein quality, and rapidly restoring popability in subsequent inbred lines due to their selectively high level of vitreous endosperm (Figure 1; Ren et al., 2018).

### Improved Agronomics of Quality Protein Popcorn Hybrids

Multiple QPP inbreds with different pedigrees were maintained throughout breeding to enable hybrid production (Table 1). Though inbreds have elevated lysine levels due to the successful introgression of the *opaque-2* allele and adequate popability, poor agronomics due to inbreeding depression, a common phenomenon in maize, disqualified the lines’ capability for commercialization as inbreds. Once hybridized, we clearly observed agronomic heterosis in QPP crosses that increased overall ear weight while maintaining popcorn-like kernels (vitreous and small). QPP hybrids had a significantly higher germination rate, number of harvested ears, ear length, number of rows per ear, and ear weight compared to the original popcorn parental inbred lines (Figure 3). Comparing QPP inbreds to popcorn inbreds, QPP inbreds had significantly longer ears and more kernel rows per ear, though 100-grain weight and ear weight were insignificantly different. Since original popcorn hybrids weren’t required in this preliminary pre-screening, it cannot be certainly ascertained if QPP hybrids are superior in agronomics compared to original popcorn hybrids. The main aim of our Quality Protein Popcorn breeding program, the improvement in protein quality in QPP inbreds and hybrids, was able to be tested and confirmed at this point in our study. However, the selection of agronomic traits from the original QPM parent and kernel traits from the original popcorn parent suggests agronomically superior popcorn varieties, an assumption that will be tested in the upcoming field season.

Multiple previous maize breeding experiments have found correlations between plant, ear, and kernel agronomic traits (Yousuf and Saleem, 2001; Ross, 2002; Malik et al., 2004; Rafiq et al., 2010). Similar to the correlations observed in our field trials, other studies have observed highly positive associations between overall grain yield, ear weight, 100-grain weight, number of rows per ear, and ear length, while other studies have suggested insignificant or negative correlations between some of these traits (Dass et al., 1990; Djordjevic and Ivanovic, 1996; Mandefro, 1998; Vasic et al., 2001; Hadji, 2004; Li et al., 2007; Yusuf, 2010; Bekele and Rao, 2014; Tulu, 2014; Ribeiro et al., 2016). Though conflicting results as to the nature and extent of agronomic correlations are not difficult to find in the literature, our study supported the prevailing notion of moderately positive correlations between ear and yield traits. Likewise, correlations found in this study between expansion volume and agronomic traits were negative, as has been observed multiple times (Brunson, 1937; Dofing et al., 1991; Ziegler and Ashman, 1994; Pereira and Amaral Júnior, 2001; Daros et al., 2002; Li et al., 2002, 2007, 2008, 2009; Dhliwayo, 2008). The genetic repeatability estimate for 100-grain weight was found at 0.683, a similar estimate to that found previously (Spaner et al., 1992). Likewise, the genetic repeatability estimate for EV was 0.582, in agreement with previous studies suggesting heritabilities of 0.61, 0.59, and 0.58 (Vasic et al., 2001; Coimbra et al., 2002; Supplementary Table S3). The correlation and heritability agreement between our values and those previously observed provided confidence that, despite the occurrence of high variance on few traits, values were suitable for evaluation and downstream analysis and QPP hybrid selection (Table 2, Supplementary Table S3, and Figure 4). High correlations and heritabilities between ear weight and ear length coupled to strong correlations with 100-grain weight suggest that future trait analysis may only require measuring one value. The measurement of ear length as a representative agronomic trait in small-scale breeding analysis may be practical and efficient, especially considering the high genetic repeatability and low standard error observed in this study. Moreover, the prevailing, significant negative relationships between popcorn quality traits and all other agronomic traits suggests that selecting for EL and vitreousness may be a tangible, successful option to improve dent by popcorn cross agronomics while maintaining popcorn quality traits.

### QPP Hybrid Evaluation and Ranking

In our approach, we hypothesized that the preliminary screening of hybrids would provide adequate information to simultaneously estimate inbred and hybrid general and specific combining abilities and improve our hybrid ranking and intermediate selection through evaluating both hybrid and inbred potential. The elucidation of parental values proved to be valuable when our ranking system’s best hybrids held very similar pedigrees. To maintain germplasm diversity in future stages of selection, representative hybrids from similar crosses were chosen based on parental breeding values. As shown in Table 2, maternal parents 9 and 10 held higher agronomic combining abilities while paternal parents 5 and 6 suggested superior popcorn quality trait combining abilities. These values aided in determining the final selection of Hybrids 28 and 38 over their reciprocals Hybrids 19 and 9, respectively. We also recognized that the use of hybrid phenotypes to suggest inbred potential did not account for poor agronomics due to inbred depression. QPP Inbreds 7 and 8 have characteristically poor seed set and slightly retained dent kernel phenotype. However, both inbreds performed well as paternal parents for Hybrids 17 and 30 and no QPP hybrid displayed a dent kernel phenotype. The utilization of hybrid analysis for inbred potential enabled the superior hybrid expression of inferior inbred lines like Inbreds 7 and 8. The high ranking of Hybrids 17 and 30 demonstrated this advantage. In other commonly used breeding selection methods, such as recurrent selection, these inferior inbreds would have been selected against in the first year of the original selection cycle (Allard, 1960).

With analysis and selection of the best QPP hybrids as the primary goal in this analysis, we also explored the basic and applied aspects of heterosis within our 44 hybrids with respect to their genetic relationships. The pedigrees and probable genetic architectures of each QPP inbred line is well-understood (Table 1). Hybrids with the same popcorn and QPM parental lines were named “Pseudo-selfed” to describe the only available interaction of the same QPM and popcorn genomes. A double back-cross of the popcorn parent suggests an 87.5:12.5 ratio of popcorn:QPM genome in the BC_2_ lines. Five generations of selfing and marker-assisted and phenotypic selection of QPM genes and QPM and popcorn traits also warrants the probable homozygosity of a majority of the introgressed QPM genome, at minimum surrounding the *opaque-2* gene on Chromosome 7 and essential *o2* modifiers, when related lines are crossed (Holding et al., 2008, 2011; Babu et al., 2015). Thus, Hybrids 5, 16, 31, and 42 were categorically grouped as “Pseudo-selfed” to describe the limited genetic diversity and interaction (Table 1). The hybrids with the “Same Popcorn Background” were assumed to have more similar genetic composition than inbreds with the “Same QPM Background” since inbreds were back-crossed twice to the original popcorn parent (Ren et al., 2018). Hybrids without similarity in either popcorn or QPM parents were further subdivided into “Same Popcorn Heterotic Pool” and “Different Heterotic Pool” categories. Popcorn Parents 2 and 3 are from the same heterotic pool, thus Hybrids 3, 4, 10, 11, 14, 15, 21, and 22 were categorized as hypothetically lesser in heterotic capacity than the rest of the hybrids interacting from different pools. Overall, these five groups of hybrids were tested for significant differences in agronomic trait values, and we observed a gradual trend in improved agronomics as groups became more genetically diverse (Figure 5). The most notable example of this gradual, step-wise trait improvement was observed in the number of ears harvested per row, followed by 100-grain weight (Figures 5A,B). The increased grain weight for QPP hybrids in different heterotic groups compared to hybrids in the same QPM background is more meaningful in light of inbred comparison, in that 100 grain weight values for QPM inbreds were significantly higher than all popcorn related lines (Figures 3E, 5B). This comparison demonstrated the efficacy of heterotic group delineation (Figures 3E, 5B). The significant improvement in ear length of hybrids with the same QPM background was surprising since QPM inbreds exhibited the shortest ears across all lines planted, and it may be an effect of extraneously improved plant agronomics in QPM dent corn backgrounds compared to popcorn backgrounds (Figures 3C, 5C). The significant drag in ear length and number of kernel rows per ear in popcorn related lines attested to the primary selection of expansion volume over the course of popcorn breeding rather than agronomic capacity, and significant improvement in these traits was observed once lines were hybridized from different heterotic groups. Overall, this empirical trend supports the theory that heterosis is manifest on a genetic basic and the degree of expression is largely determined by genetic relatedness of the parents (Moll et al., 1965; Reif et al., 2003, 2005; Springer and Stupar, 2007; Fu et al., 2014). However, this progression of improvement was only observed for agronomic traits. Expansion volume and popability values in more popcorn-related lines were superior to those of unrelated pedigrees. Additionally, lysine contents of QPP crosses compared to those of their respective parents suggested an additive effect (Figure 8C). Though the underlying causes of these heterotic patterns have yet to be elucidated, grouping hybrids and observing this agronomic trend aided our eventual selection of hybrids to favor the “complete hybrid” group.

Overall, these genetic analyses were used alongside a tailored ranking system for QPP hybrid selection. While selection indices are more commonly used for recurrent inbred selection, it was evident that a model was needed for our hybrid analysis. Such a model could properly manipulate the genetic potentials of multiple traits into a single sum that could accurately represent hybrid value (Tardin et al., 2007; Marinho et al., 2014). The ranking system utilized is similar to a Rank Summation Index in which each trait is evaluated across hybrids, ranked independently, and then summed for a final ranking value (Mulamba and Mock, 1978; Figure 6). In our model, the economic value of each trait was partitioned through selection intensity coefficients and the genetic value was imputed through trait value and standard deviation (Table 4). This allowed for both an overall hybrid rank and the partitioning of rank value by trait, a distinction from other ranking systems (Figure 7). This simple model agreed well with concurrent analyses of our hybrids’ genetic potential and elite hybrids were narrowed quickly. Due to Inbreds 9 and 10 having superior maternal agronomic capabilities, Hybrids 28 and 38 were chosen for continued analysis instead of their reciprocals. Hybrid 20 was also selected since it ranked well and the agronomic pGCAs for Inbred 10 were high. Hybrid 43 came from a relatively more diverse cross (Inbred 10 × Inbred 11), and notably had a consistent mushroom flake type (Figure 6). Popcorn hybrid flake types are commonly classified as either mushroom or butterfly (Eldredge and Thomas, 1959). Butterfly hybrid seed are commonly selected for packaging and can further be classified as unilateral, bilateral, or multilateral depending on the number and symmetry of flake branching, while popped mushroom hybrids are preferred as marketable products due to the minimized breakage during coating and packaging (Eldredge and Thomas, 1959; Sweley et al., 2011). This distinction in popped flake morphology compared to the other elite hybrids made Hybrid 43 a top contender for further analysis. Finally, to sustain diversity, Hybrids 30, 25, and 17 were considered for advancement. During this portion of analysis the relatively lower broad-sense heritability estimates, or the proportion of total phenotypic variance due to additive, dominant, and epistatic genetic effects, for inbred lines contrasted with higher repeatability estimates for SCA. Due to the use of hybrids to estimate inbred heritability including non-additive effects, it is reasonable that SCA estimates had higher genetic repeatability and lower standard error. Moreover, since hybrids were being evaluated, all genetic effects were considered applicable for selection and SCA values became paramount in the selection of elite hybrids (Supplementary Table S3). The highest repeatability estimates were identified for ear length and ear weight, though ear weight had a very high standard error. Both of these agronomic traits estimated high SCA values for Hybrid 25 compared to Hybrids 17 and 30, albeit not significant (Supplementary Table S3). Expansion Volume SCAs for Hybrids 17 and 30 were superior to Hybrid 25 (0.582 repeatability with high standard error), but Hybrid 25 held a significantly better 100-grain weight (0.683 repeatability) and significantly larger kernel size (0.676 repeatability) compared to these two hybrids (Supplementary Table S3). Hybrids 17 and 30 also included Inbreds 7 and 8 as paternal parents; QPP inbreds that were difficult to advance due to low inbred grain fill and sustained dent kernel phenotype. Hybrid 25 received low index sums for all traits except rot susceptibility, a less valuable trait outweighed by other highly-correlative traits to grain yield. Therefore, Hybrid 25 was ultimately selected for continued analysis. Other top hybrids had notable SCA values in agronomic and popcorn quality traits. Hybrids 20 and 28 held positive 2.6 and 2.7 (cm) values for SCA in ear length, Hybrid 43 had the highest SCA value for number of kernel rows per ear (2.265 rows, 0.673 repeatability), and Hybrids 20, 28, and 38 all had significantly large SCA values for expansion volume, estimated at 50.11, 48.94, and 57.98 mL/20 g, respectively (Supplementary Table S3). Due to superior agronomics and confirmed quality protein, as further described, Hybrids 20, 25, 28, 38, and 43 were chosen for continued analysis.

**TABLE 4:** Relative trait weighting values for ranking model.

**Trait**	**Weight value (I_i_)**
Germination rate (%)	0.7
Days to pollination (days)	0
Pest/rot susceptibility	0.5
Number of ears harvested	0.6
Ear length (cm)	0.5
Number of rows per ear	0.4
Ear weight (g)	0.8
Kernel size	0
100-Grain weight (g)	0.7
Vitreousness	0.6
Pop-ability	0.85
Expansion volume (mL/g)	0.85

*Traits were ranked according to economic value with scores ranging from 0 to 1 in increasing importance. Popcorn quality traits were ranked highest followed by yield and agronomic traits. Number of Days to Pollination was not used to determine rank since it held minimal economic value. Kernel size was a repetitive measure of 100-grain weight and was not used to rank hybrids.*

### Elevated Lysine Content in QPP Hybrids Across Popping Methods

In conjunction with hybrid selection through agronomic and popping evaluations, 10 hybrids were chosen for amino acid profiling of free and protein-bound amino acids in the kernel. Previous temporal studies on maize endosperm protein quality have observed that lysine and tryptophan amino acid levels differentially decrease during kernel maturity with high variability between genetic backgrounds (Sethi et al., 2020). However, tryptophan and lysine levels within a genetic background correlate in relative abundance (Hernandez and Bates, 1969; Krivanek et al., 2007; Olakojo et al., 2007). Therefore, acidic hydrolysis, which destroys tryptophan, was conducted for protein-bound lysine determination. All free amino acids including tryptophan were recovered and measurable. Principle Component Analyses on protein-bound and free amino acid data demonstrated that the QPP proteome imitated that of QPM rather than the genetically dominating popcorn background (Figure 8A and Supplementary Figure S4). Genetic repeatability estimates including both additive and non-additive effects were calculated per genotype for raw kernel protein-bound amino acids. Eight out of the sixteen amino acids had high repeatability estimates above 0.700 (excluding isoleucine at 0.693), including lysine, histidine, leucine, methionine, and phenylalanine essential amino acids. The high repeatability measurement for lysine validated downstream selection for elevated levels. Ground raw kernel powder of the 10 best QPP hybrids revealed an average 1.45-fold increase in protein-bound lysine, and the five selected QPP hybrids exhibited an average 1.52-fold increase in protein-bound lysine compared to popcorn germplasm (Supplementary Table S1). These fold changes of increased lysine were, similarly, observed by Ren et al. with QPP inbreds, ranging from a 1.45–2.0-fold increase in the amino acid abundance compared to original popcorn inbreds (Ren et al., 2018). The Food and Agriculture Organization of the United Nations recommends a 5.8% lysine requirement in total protein for children ages 2–5 for optimum health. During QPM hybrid production, QPM inbred pools conferred 2.7–4.5% lysine in total protein, an improvement from 1.6 to 2.6% in normal maize and considered an acceptable standard for “Quality Protein” Maize. In this study, protein-bound lysine accounted for ∼4.65% of total protein in QPP hybrids compared to ∼2.65% in popcorn inbreds and surpassed the previously cited range for QPM breeding pools (Vasal, 2002; Krivanek et al., 2007; Supplementary Table S1).

Additionally throughout CIMMYT’s breeding of QPM, researchers understood the necessity of monitoring the lysine and tryptophan content of raw, whole grain flour and consumable products such as nixtamal, masa, and tortillas. After quantification, researchers found an overall significant decrease in tryptophan and both significant and insignificant losses of lysine in all consumable products (Ortega et al., 1986). However, this trend was general to all tested maize lines and QPM was legitimized as effective in conferring elevated lysine and tryptophan levels in the cooked, consumable products (Ortega et al., 1986). Since popcorn is consumed by humans after popping, popped flake amino acid levels were of paramount importance to evaluate and measurements are sparse in the literature. The last available amino acid profile of oil- and air- popped popcorn was in 1991 (Cutrufelli, 1991). Popping effect on amino acid content, correlations between raw kernel flour and that of popped flakes, and specific effect of each popping mechanism have remained unexplored. Analysis on popped flakes revealed a general trend in free amino acid level decrease, while protein-bound amino acid fluctuations were dependent on the residue. Histidine, isoleucine, leucine, lysine, methionine, phenylalanine, threonine, and valine are considered essential amino acids because they are not synthesized by the human body in adequate amounts for maintained human health (Wu, 2009). After popping by air or microwave methods, all quantified essential amino acids except lysine and methionine increased in protein-bound abundance compared to raw kernel flour while oil-popped flakes decreased the abundance of all protein-bound amino acids, though confidence intervals overlapped (Supplementary Tables S1, S4–S6). These results suggest that air and microwave popping may not affect amino acid composition or abundance as severely as oil popped methods. Furthermore, protein-bound lysine was the only essential amino acid to significantly decrease after popping (Supplementary Tables S1, S4–S6). With lysine already the most limiting amino acid in maize grain, this observation reinforced the requirement for elevated lysine in the popcorn kernel to convey higher abundance in the popped flake (Alan, 2009). The increase in both lysine and tryptophan abundance compared to popcorn parents, maintained before and after popping by various methods, ultimately validated the proteomic biofortification of the Quality Protein Popcorn endosperm in its raw and popped form. On average, QPP air popped flakes offered more lysine than original popcorn parent raw kernel flour and approximately two times more lysine than original parent air popped flakes. In context, the recommended intake of lysine is ∼30 mg per kilogram of body weight per day, which converts to ~2.108 g per day for a 68 kg (150 pound) individual (Elango et al., 2009). Microwavable popcorn packets use ∼47 grams of popcorn kernels per bag. When air popped, one bag of QPP hybrids would fulfill ∼8.6% of lysine daily dietary requirement while original popcorn parents would only satisfy ∼4.3% (Supplementary Tables S4, S7).

With these raw and popped kernel amino acid values, we are confident that QPP hybrids are successfully yielding the characteristic *opaque-2* endosperm proteome while maintaining popability and improving popcorn agronomics. As introgressing dent germplasm into popcorn has been previously difficult, we suggest a prerequisite phenotype of highly vitreous dent endosperm for future dent by popcorn crosses that aim to restore and maintain popcorn quality traits. This phenotype was key for rapid restoration of QPP popability. Once at the inbred stage, hybrid production and analysis of QPP lines was necessary to improve agronomics. The integration of inbred and hybrid analysis proved helpful in the final determination of our elite QPP hybrids and is transferable to various other breeding programs involved in hybrid testing and selection. Now that the five most elite QPP hybrids have been selected, it is necessary to determine how these crosses compare to currently marketed popcorn varieties in agronomic and quality traits and this analysis is currently underway.

## Data Availability Statement

All data generated from this study (excluding proprietary popcorn identities) is available upon request. R scripts for all genetic analysis and the ranking model are available upon request: dholding2@unl.edu or lmarshall3@unl.edu.

## Author Contributions

LP and DH designed the research and wrote the manuscript. LP, AY, RA, and DH analyzed the data. All authors performed the research.

## Conflict of Interest

The authors declare that this study received funding from ConAgra Brands^®^. Moreover, OR was employed by the company ConAgra Brands^®^. The funder was not involved in the study design, collection, analysis, interpretation of data, the writing of this article or the decision to submit it for publication.

The remaining authors declare that the research was conducted in the absence of any commercial or financial relationships that could be construed as a potential conflict of interest.
