# Supplementary material for: Production and Selection of Quality Protein Popcorn Hybrids Using a Novel Ranking System and Combining Ability Estimates
Source: Front Plant Sci. 2020 Jun 23;11:698. doi: 10.3389/fpls.2020.00698 (PMC7325744; doi:10.3389/fpls.2020.00698)
Supplement: Supplementary file 1 [file Data_Sheet_1.PDF]

Supplementary Material

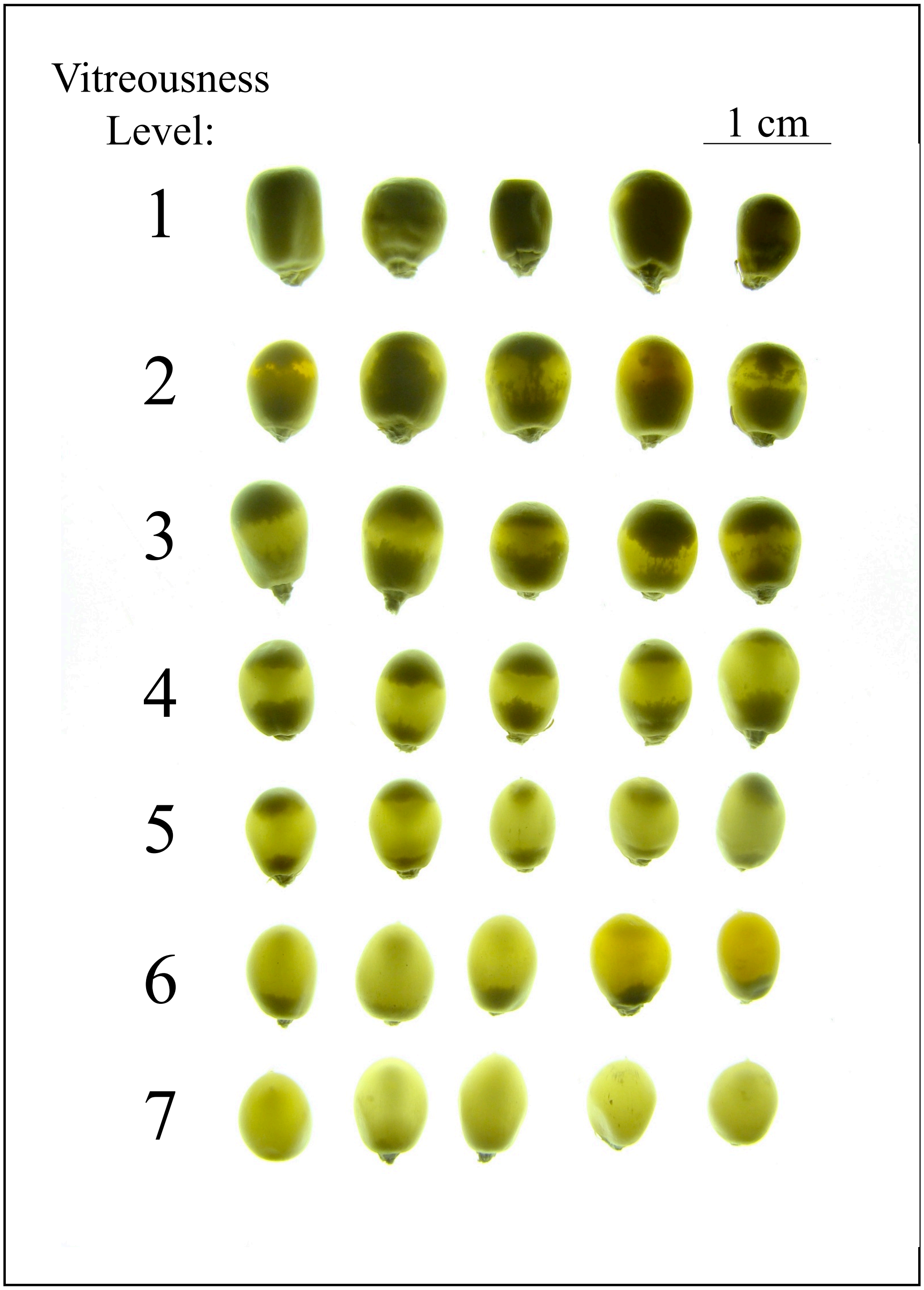

Supplementary Figure 1 | Popcorn kernel endosperm vitreousness scale. Ten grams of kernels were randomly selected from each row of the 2019 field and scored on a continuous scale of 1-7, with a rank of '1' being nearly complete opacity and '7' as completely vitreous.

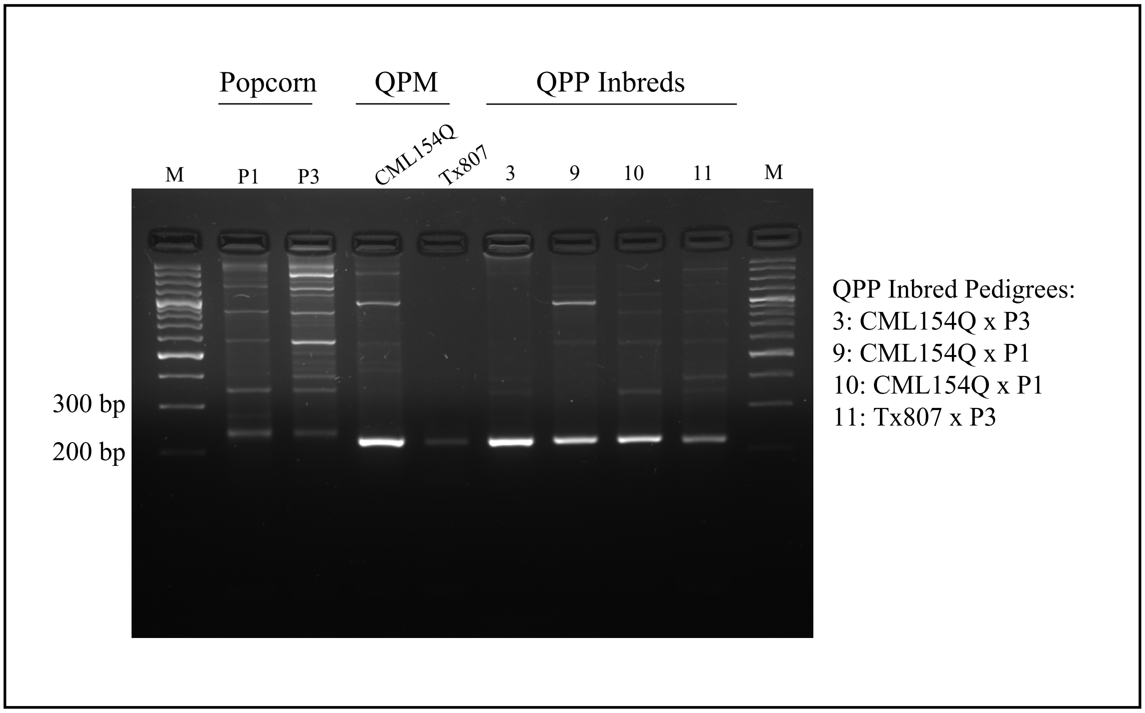

Supplementary Figure 2 | DNA-based marker aided verification of *o2o2* genotype in parental inbreds. All QPP inbred parents were genotyped with *opaque-2* in-gene marker umc1066 and/or flanking marker bnlg1200. As shown, popcorn parents encode a differentiated, wild-type *opaque2* allele while QPM parents have a lower band. All QPP inbreds shown are crosses between Popcorn Parent 1, Popcorn Parent 3, and CML154Q and Tx807. All inbreds displayed the alike lower band to QPM parents.

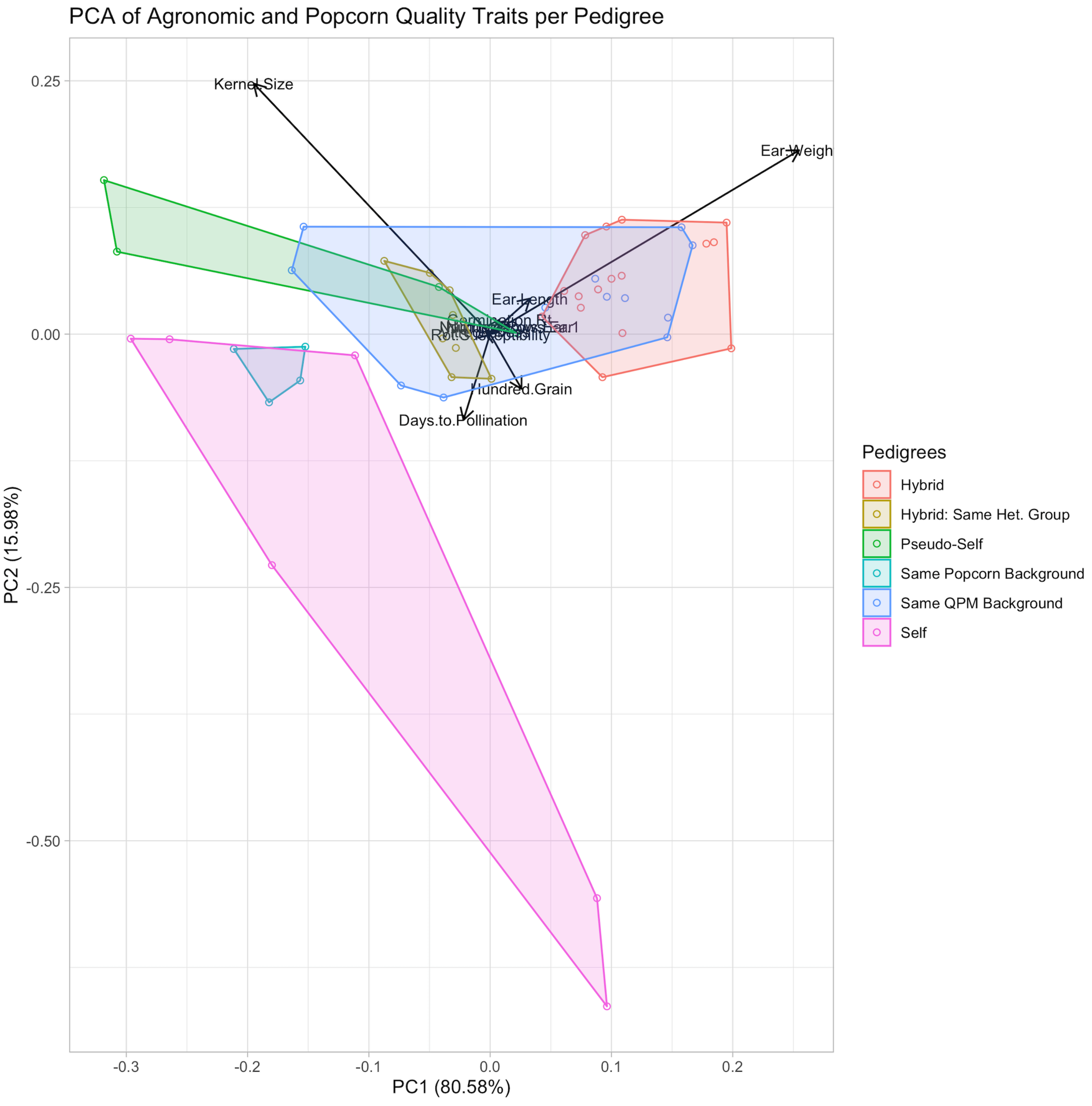

Supplementary Figure 3 | Principle Component Analysis of QPP Hybrids, Inbreds, QPM, and Popcorn Parents Grown in 2019 fields. Principle Component scores (PC1 and PC2) from each variable are described as text in plot. Six clusters of pedigree categories (Self, Pseudo-self, Same Popcorn Background, Same QPM Background, Hybrid: Same Het. Group, and Hybrid) were observed.

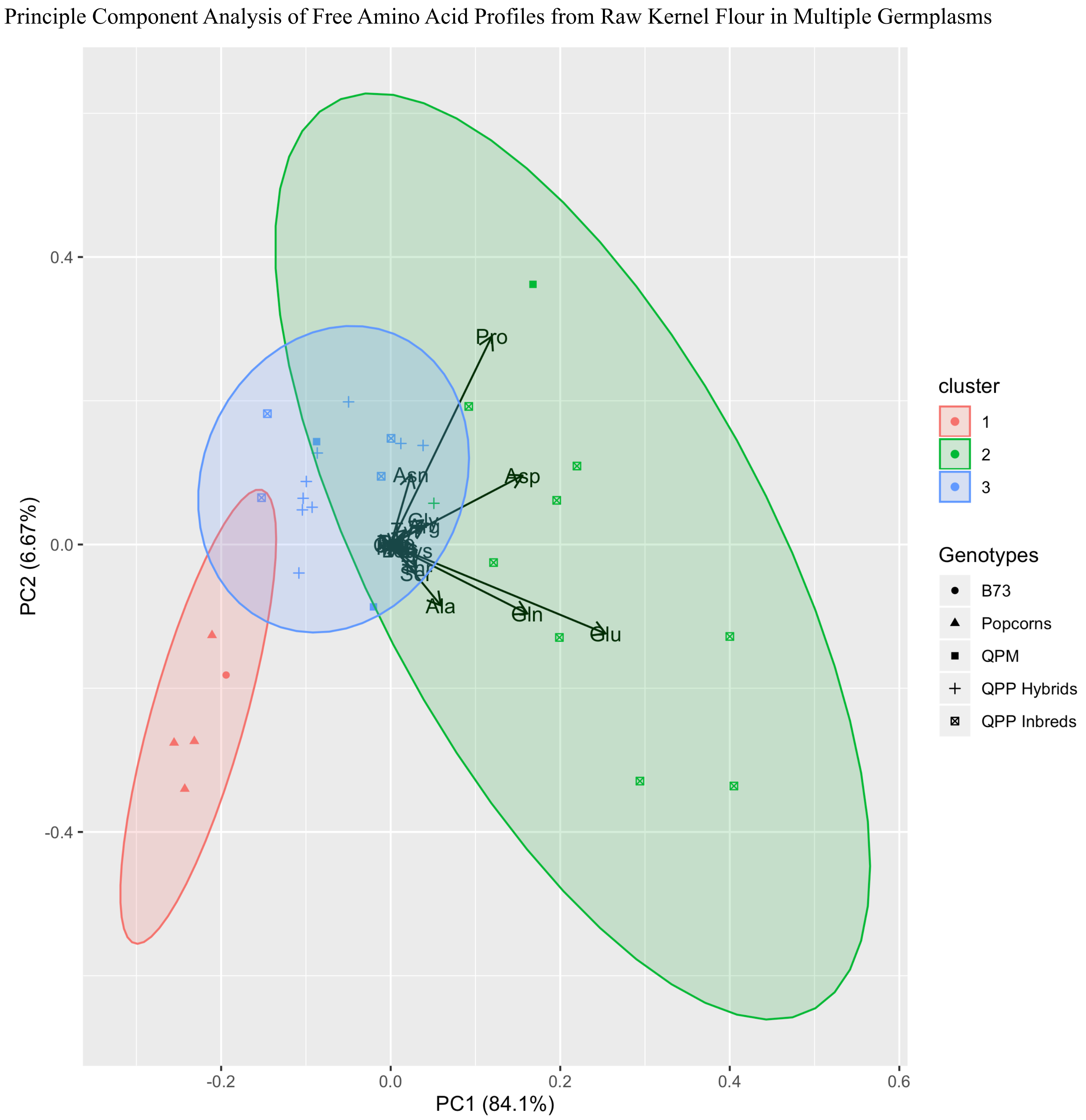

Supplementary Figure 4 | Principle Component Analysis of Free Amino Acids from raw Kernel Flour in Multiple Germplasms. All amino acids were available for quantification in free form. Three clusters arose from the data; one of popcorn parents (red), one of QPP hybrids (blue), and one of QPP inbreds (green). Inbreds were characterized with higher proline, aspartate, glutamate, and glutamine levels. QPP hybrids overlapped with both clusters though most overlay occurred between QPP Inbreds and Hybrids. QPM inbreds were present in both QPP inbred and hybrid clusters.

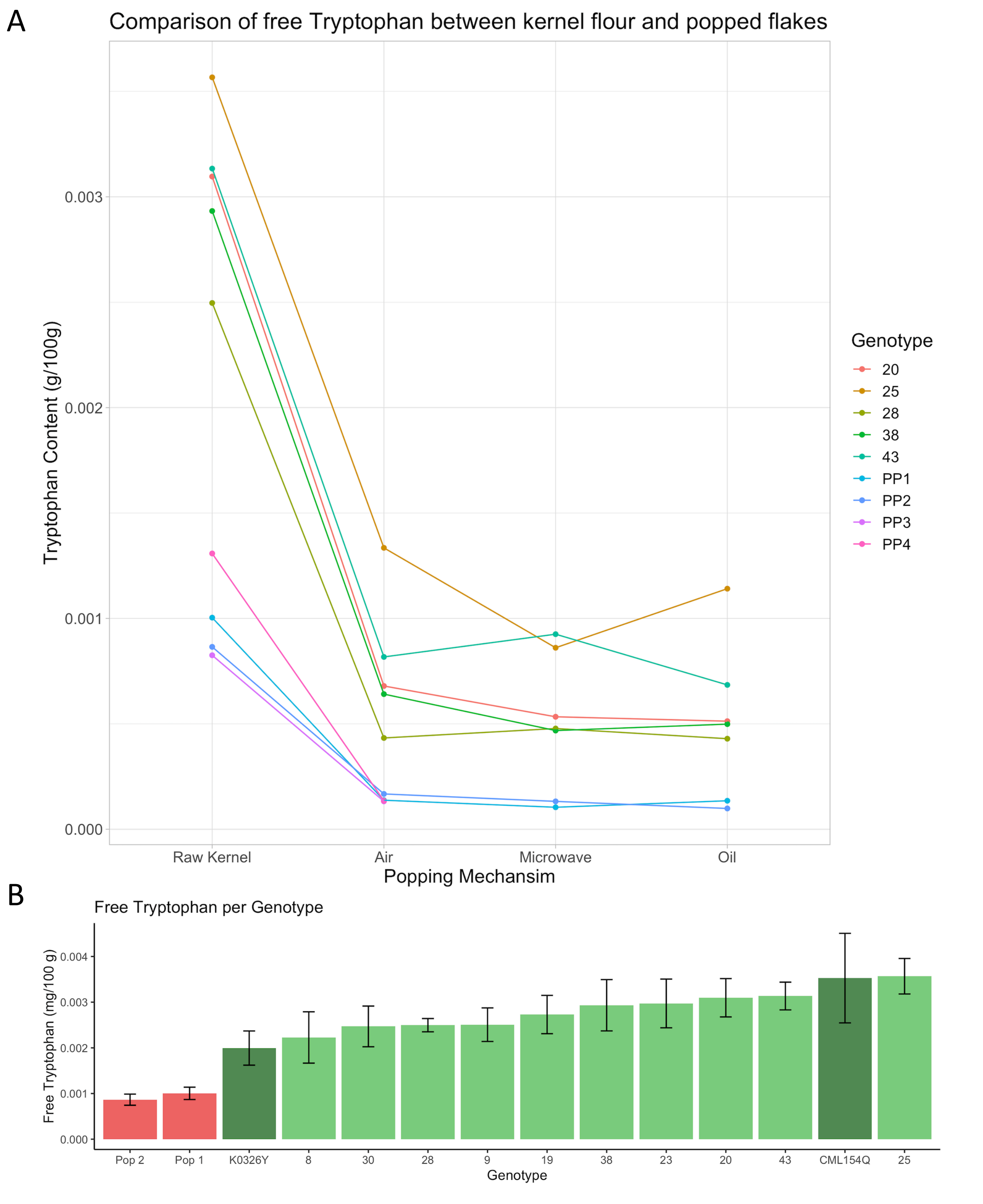

Supplementary Figure 5 | Free Tryptophan Values and Effect of Popping Methods. **(A)** Alike to protein-bound and free lysine, free tryptophan values from raw kernel flour decreased at a similar rate when popped by multiple methods and correlation coefficients were high (range of 0.882 – 0.992). **(B)** All QPP hybrids (light green) held larger raw kernel flour free-tryptophan values than popcorn parents (red) and potentially QPM parents (dark green). At minimum, QPP hybrids were insignificantly different in free tryptophan content than QPM parents.

Genotype	Ala	Arg	Asx	Glx	Gly	His	Ile	Leu	Lys	Met	Phe	Pro	Ser	Thr	Tyr	Val
Popcorn Parent 1	1.101±0.052	0.358±0.023	0.793±0.056	2.751±0.075	0.952±0.08	0.452±0.014	0.647±0.044	2.084±0.123	0.372±0.026	0.257±0.021	0.71±0.014	1.267±0.058	0.692±0.03	0.589±0.049	0.461±0.034	0.557±0.02
Popcorn Parent 2	0.963±0.126	0.362±0.025	0.664±0.047	2.449±0.201	0.886±0.115	0.394±0.015	0.58±0.059	1.852±0.177	0.326±0.018	0.194±0.02	0.652±0.051	1.1±0.069	0.638±0.061	0.5±0.042	0.379±0.035	0.498±0.032
Popcorn Parent 3	0.996±0.012	0.354±0.002	0.685±0.006	2.446±0.048	0.804±0.009	0.385±0.015	0.563±0.004	1.848±0.042	0.415±0.015	0.196±0.004	0.667±0.027	1.216±0.003	0.658±0	0.591±0.034	0.413±0.037	0.5±0
Popcorn Parent 4	1.385±0.035	0.402±0.017	0.861±0.002	3.145±0.189	1.222±0.035	0.458±0.017	0.802±0.02	2.617±0.171	0.363±0.049	0.236±0.009	0.817±0.05	1.321±0.077	0.832±0.04	0.628±0.038	0.574±0.008	0.662±0.012
B73	0.92±0.041	0.46±0.02	0.735±0.041	2.369±0.108	0.924±0.07	0.439±0.017	0.554±0.041	1.65±0.096	0.457±0.033	0.209±0.007	0.636±0.025	1.078±0.054	0.64±0.023	0.523±0.031	0.373±0.006	0.538±0.028
CML154Q	0.754±0.058	0.57±0.033	1.098±0.133	2.233±0.161	0.93±0.029	0.541±0.026	0.509±0.051	1.187±0.173	0.629±0.022	0.153±0.007	0.557±0.037	1.183±0.102	0.586±0.04	0.548±0.04	0.311±0.034	0.6±0.007
K0326Y	0.751±0.107	0.585±0.057	0.955±0.185	2.16±0.213	0.866±0.125	0.576±0.024	0.487±0.047	1.257±0.141	0.589±0.066	0.166±0.013	0.545±0.05	1.168±0.088	0.611±0.069	0.543±0.062	0.307±0.023	0.603±0.052
Tx807	0.874±0.109	0.632±0.046	1.496±0.026	2.447±0.097	0.99±0.032	0.571±0.019	0.609±0.059	1.56±0.231	0.667±0.002	0.15±0.009	0.663±0.046	1.132±0.08	0.689±0.053	0.604±0.049	0.351±0.016	0.63±0.023
QPP Inbred 1	0.674±0.05	0.401±0.045	0.967±0.026	2.247±0.043	0.876±0.104	0.527±0.031	0.448±0.034	1.166±0.115	0.503±0.018	0.122±0.011	0.503±0.033	1.12±0.032	0.518±0.024	0.483±0.009	0.302±0.034	0.53±0.042
QPP Inbred 2	0.57±0.033	0.489±0.046	0.869±0.151	1.828±0.158	0.724±0.076	0.486±0.015	0.378±0.027	0.919±0.112	0.502±0.044	0.111±0.005	0.446±0.064	0.98±0.05	0.485±0.042	0.43±0.051	0.251±0.011	0.488±0.031
QPP Inbred 3	0.722±0.071	0.449±0.038	0.985±0.208	2.258±0.011	0.797±0.052	0.504±0.037	0.457±0.062	1.052±0.16	0.557±0.06	0.133±0.005	0.49±0.073	1.014±0.003	0.53±0.065	0.531±0.047	0.274±0.012	0.538±0.051
QPP Inbred 4	0.67±0.066	0.454±0.001	0.908±0.053	2.33±0.044	0.935±0.036	0.486±0.017	0.424±0.031	0.969±0.172	0.591±0.04	0.14±0.006	0.463±0.013	1.059±0.085	0.521±0.033	0.523±0.005	0.293±0	0.527±0.014
QPP Inbred 5	0.599±0.045	0.427±0.011	0.8±0.014	2.07±0.083	0.803±0.027	0.523±0.014	0.414±0.05	1.073±0.135	0.491±0.008	0.11±0.004	0.476±0.063	1.131±0.049	0.504±0.042	0.467±0.028	0.273±0.052	0.52±0.015
QPP Inbred 6	0.684±0.089	0.448±0.036	0.801±0.011	2.016±0.02	0.803±0.071	0.546±0.012	0.47±0.034	1.128±0.055	0.563±0.047	0.104±0.005	0.514±0.062	1.167±0.017	0.539±0.033	0.497±0.046	0.309±0.023	0.564±0.041
QPP Inbred 7	1.045±0.031	0.546±0.024	1.512±0.033	2.847±0.074	1.13±0.105	0.539±0.016	0.62±0.013	1.674±0.024	0.61±0.007	0.127±0	0.698±0	1.211±0.029	0.696±0.033	0.622±0.005	0.386±0.039	0.641±0.002
QPP Inbred 8	0.951±0.087	0.638±0.017	1.27±0.021	2.603±0.158	1.033±0.091	0.606±0.023	0.656±0.068	1.726±0.251	0.65±0.02	0.154±0.002	0.692±0.102	1.367±0.075	0.73±0.114	0.646±0.058	0.392±0.074	0.717±0.036
QPP Inbred 9	0.617±0.065	0.424±0.055	0.64±0.007	1.766±0.132	0.782±0.009	0.512±0.037	0.414±0.026	1.034±0.103	0.485±0.018	0.106±0.014	0.451±0.034	1.027±0.093	0.485±0.046	0.461±0.027	0.274±0.015	0.525±0.038
QPP Inbred 10	0.643±0.023	0.454±0.066	0.785±0.039	1.906±0.115	0.867±0.018	0.525±0.026	0.48±0.022	1.155±0.05	0.551±0.016	0.132±0.006	0.534±0.04	1.045±0.035	0.523±0.022	0.471±0.034	0.327±0.022	0.561±0.038
QPP Inbred 11	0.636±0.016	0.496±0.064	0.888±0.149	2.049±0.041	0.736±0.071	0.49±0.004	0.436±0.001	1.046±0.028	0.644±0.04	0.131±0.005	0.484±0.022	1.112±0.02	0.527±0.017	0.584±0.011	0.296±0.018	0.541±0.012
QPP Inbred 12	0.843±0.021	0.488±0.032	0.88±0.056	2.311±0.06	0.87±0.035	0.535±0.006	0.49±0.001	1.258±0.026	0.635±0.015	0.141±0	0.548±0.023	1.192±0.018	0.603±0.009	0.596±0.001	0.353±0.012	0.605±0.001
QPP Hybrid 8	0.634±0.048	0.457±0.029	0.805±0.101	1.911±0.049	0.826±0.121	0.562±0.049	0.417±0.026	1.114±0.063	0.477±0.017	0.125±0.015	0.495±0.028	1.055±0.065	0.505±0.031	0.472±0.023	0.316±0.033	0.521±0.024
QPP Hybrid 9	0.667±0.039	0.444±0.057	0.865±0.087	1.972±0.083	0.856±0.103	0.535±0.028	0.451±0.027	1.202±0.063	0.469±0.045	0.128±0.019	0.525±0.026	1.051±0.039	0.52±0.033	0.483±0.027	0.309±0.026	0.529±0.029
QPP Hybrid 19	0.708±0.12	0.498±0.088	0.868±0.204	2.005±0.23	0.828±0.117	0.577±0.047	0.474±0.074	1.192±0.185	0.525±0.077	0.126±0.013	0.536±0.071	1.097±0.063	0.548±0.07	0.496±0.069	0.322±0.022	0.563±0.06
QPP Hybrid 20	0.73±0.065	0.513±0.058	0.981±0.137	2.128±0.117	0.946±0.045	0.582±0.037	0.512±0.036	1.327±0.082	0.558±0.047	0.138±0.017	0.571±0.026	1.136±0.057	0.591±0.031	0.54±0.033	0.331±0.012	0.583±0.04
QPP Hybrid 23	0.67±0.049	0.469±0.021	1.001±0.199	2.073±0.163	0.85±0.054	0.534±0.014	0.457±0.028	1.165±0.083	0.504±0.014	0.124±0.006	0.519±0.036	1.082±0.022	0.532±0.023	0.476±0.018	0.299±0.018	0.532±0.013
QPP Hybrid 25	0.624±0.014	0.517±0.04	0.895±0.135	1.998±0.074	0.839±0.084	0.548±0.03	0.421±0.011	1.03±0.032	0.536±0.042	0.132±0.012	0.474±0.012	1.059±0.05	0.513±0.035	0.482±0.024	0.293±0.016	0.542±0.024
QPP Hybrid 28	0.666±0.072	0.493±0.072	0.839±0.169	2.041±0.242	0.813±0.143	0.57±0.035	0.474±0.053	1.223±0.154	0.524±0.047	0.119±0.021	0.529±0.071	1.126±0.088	0.559±0.064	0.51±0.051	0.286±0.045	0.565±0.052
QPP Hybrid 30	0.705±0.065	0.509±0.041	0.813±0.094	2.048±0.098	0.817±0.01	0.542±0.035	0.472±0.025	1.214±0.106	0.539±0.039	0.143±0.022	0.534±0.029	1.121±0.076	0.557±0.04	0.528±0.034	0.307±0.024	0.562±0.029
QPP Hybrid 38	0.71±0.053	0.528±0.035	0.964±0.171	2.061±0.148	0.9±0.116	0.555±0.029	0.478±0.04	1.217±0.106	0.552±0.024	0.137±0.014	0.54±0.037	1.107±0.061	0.558±0.048	0.527±0.03	0.29±0.032	0.571±0.038
QPP Hybrid 43	0.702±0.067	0.51±0.046	0.97±0.186	2.073±0.131	0.819±0.087	0.52±0.017	0.474±0.022	1.175±0.072	0.589±0.03	0.149±0.019	0.533±0.033	1.109±0.044	0.546±0.023	0.55±0.04	0.301±0.022	0.55±0.013

Supplementary Table 1: Protein-Bound Amino Acid Values (g/100g) in Raw Kernel Flour. Protein-bound amino acid values of sixteen amino acids are recorded. Aspartate and asparagine (Asx), glutamine and glutamate (Glx), Serine, and Tryptophan are destroyed during acidic hydrolysis, the procedure used for amino acid quantification. Standard deviations were calculated by two-six biological replications, dependent on genotype. Lysine levels are shaded in gray.

	Ala	Arg	Asn	Asp	Gln	Glu	Gly	His	Ile	Leu
**Popcorn Parent 4**	0.0054±0.0008	0.0059±0.0004	0.0235±0.0027	0.004±0.0002	0.0001±0	0.0047±0.0002	0.0015±0.0003	0.0024±0.0003	0.0004±0.0001	0.0005±0
**Popcorn Parent 3**	0.0187±0.0011	0.0063±0.0003	0.0211±0	0.0101±0.0023	0.0031±0	0.0132±0.0037	0.0064±0.0003	0.0045±0	0.0007±0	0.001±0.0002
**Popcorn Parent 1**	0.0046±0.0016	0.0052±0.0001	0.0407±0.0034	0.0084±0.0023	0.0081±0.0061	0.0146±0.0034	0.0057±0.001	0.0045±0.0012	0.0005±0	0.0005±0
**Popcorn Parent 2**	0.0023±0.0004	0.0074±0.0015	0.0268±0.0024	0.0046±0.0013	0.0023±0.0005	0.0173±0.0025	0.0059±0.0013	0.0022±0.0005	0.0001±0	0.0002±0
**B73**	0.0072±0.0023	0.0072±0.0012	0.0295±0.0021	0.0155±0.0056	0.0035±0.001	0.0316±0.0031	0.0089±0.002	0.0029±0.001	0.0006±0.0001	0.0007±0.0001
**CML154Q**	0.0205±0.0068	0.0396±0.0019	0.0713±0.0078	0.1241±0.0088	0.0958±0.0366	0.1199±0.0146	0.0308±0.0011	0.0132±0.0026	0.0033±0.0013	0.0065±0.0032
**K0326Y**	0.0101±0.005	0.0295±0.0093	0.0704±0.0137	0.0711±0.0325	0.015±0.0153	0.0433±0.0237	0.0235±0.0073	0.0119±0.0045	0.0029±0.0026	0.0031±0.0027
**QPP Inbred 1**	0.0274±0.0198	0.0283±0.0004	0.0676±0.0059	0.1289±0.0022	0.099±0.0761	0.1588±0.053	0.0335±0.0005	0.0087±0.0019	0.0027±0.0024	0.0061±0.0046
**QPP Inbred 10**	0.0046±0.0003	0.0113±0.0014	0.0486±0.0012	0.029±0.0022	0.0047±0.0006	0.031±0.0016	0.0103±0.0004	0.0055±0.0019	0.001±0	0.0008±0
**QPP Inbred 11**	0.0339±0.0201	0.0417±0.0046	0.0443±0.0113	0.1151±0.0011	0.0787±0.0373	0.1855±0.0048	0.0434±0.0014	0.0101±0.002	0.002±0.0006	0.0055±0.0036
**QPP Inbred 12**	0.0931±0.0423	0.0346±0.0035	0.0422±0.0065	0.116±0.0058	0.0677±0.0228	0.1715±0.0277	0.0483±0.0062	0.0108±0.0004	0.0045±0	0.0133±0.0019
**QPP Inbred 2**	0.0152±0.0023	0.0292±0.0011	0.0682±0.0118	0.1067±0.0203	0.0511±0.0093	0.1248±0.0098	0.0335±0.0009	0.0078±0.0019	0.0008±0.0003	0.0023±0.0004
**QPP Inbred 3**	0.05±0.024	0.036±0.0009	0.0598±0.012	0.1332±0.0213	0.1658±0.0847	0.2007±0.0464	0.034±0.0019	0.0195±0.0006	0.0014±0.0005	0.006±0.0047
**QPP Inbred 4**	0.0532±0.0036	0.0409±0.0002	0.0548±0.008	0.1637±0.0131	0.1724±0.0445	0.2373±0.0174	0.0343±0.0002	0.019±0.0076	0.0029±0.0006	0.0102±0.0004
**QPP Inbred 5**	0.0176±0.0103	0.0336±0.0028	0.0598±0.0035	0.1047±0.0027	0.0819±0.0834	0.1398±0.0637	0.0315±0.0016	0.0114±0.0022	0.0013±0.0004	0.0034±0.0019
**QPP Inbred 6**	0.0114±0.0032	0.0354±0.0032	0.0442±0.0032	0.0766±0.0129	0.0227±0.0256	0.0949±0.0627	0.0352±0.0169	0.0093±0.002	0.002±0.0006	0.0021±0.0011
**QPP Inbred 7**	0.0637±0.0134	0.0551±0.0017	0.0667±0.0004	0.1561±0.0068	0.1082±0.0229	0.292±0.0241	0.0486±0.0003	0.0201±0.0004	0.0041±0.0003	0.0093±0.0011
**QPP Inbred 8**	0.0146±0.0035	0.0528±0.0065	0.0648±0.0049	0.0931±0.0379	0.0084±0.0092	0.0907±0.0473	0.0393±0.0082	0.0149±0.0027	0.001±0.0002	0.0012±0.0005
**QPP Inbred 9**	0.0041±0.0001	0.0182±0.0011	0.0369±0.0071	0.0214±0.0091	0.0051±0.0004	0.0362±0.0023	0.0151±0.0102	0.0048±0.0007	0.0015±0	0.0011±0
**Tx807**	0.0134±0.0011	0.0344±0.0004	0.0954±0.0035	0.1004±0.0065	0.0217±0.0085	0.0898±0.0075	0.0321±0.0007	0.0145±0.0014	0.0015±0.0002	0.0033±0.0004
**Hybrid 19**	0.0066±0.0011	0.0247±0.0058	0.0636±0.0114	0.0676±0.0189	0.0064±0.0054	0.0508±0.0228	0.0232±0.0062	0.0075±0.0022	0.0005±0.0001	0.0009±0.0004
**Hybrid 20**	0.0089±0.002	0.0235±0.0046	0.0704±0.0096	0.0721±0.0102	0.0075±0.0073	0.0535±0.0234	0.0251±0.0057	0.0077±0.0013	0.0006±0.0001	0.0008±0.0001
**Hybrid 23**	0.014±0.0036	0.0186±0.0054	0.0698±0.0079	0.1053±0.0158	0.0517±0.0423	0.0932±0.0262	0.0306±0.0011	0.0083±0.0032	0.001±0.0004	0.0028±0.0015
**Hybrid 25**	0.0167±0.009	0.0243±0.002	0.0629±0.0089	0.1068±0.0349	0.0702±0.0704	0.0932±0.0484	0.0279±0.0061	0.009±0.0023	0.0013±0.001	0.0036±0.0032
**Hybrid 28**	0.007±0.0014	0.0201±0.0059	0.0614±0.0081	0.0692±0.0253	0.0137±0.0173	0.0532±0.0281	0.0226±0.0071	0.0071±0.0015	0.0005±0	0.0009±0.0005
**Hybrid 30**	0.012±0.0044	0.0131±0.006	0.0576±0.0142	0.0688±0.0322	0.0214±0.0262	0.0648±0.036	0.0227±0.0098	0.0073±0.0022	0.0006±0.0001	0.0013±0.0008
**Hybrid 38**	0.0071±0.0023	0.0213±0.0081	0.0703±0.0122	0.0624±0.0226	0.0079±0.0089	0.0513±0.0222	0.0241±0.0074	0.0075±0.0024	0.0005±0.0001	0.0009±0.0002
**Hybrid 43**	0.0121±0.0033	0.0265±0.0063	0.0693±0.0124	0.0809±0.0207	0.0399±0.0433	0.0928±0.0404	0.0292±0.0049	0.0087±0.0021	0.0009±0.0001	0.0015±0.0005
**Hybrid 8**	0.0063±0.0012	0.0216±0.0043	0.0605±0.0081	0.0677±0.0215	0.0112±0.0101	0.0484±0.0261	0.0218±0.0081	0.0062±0.0016	0.0003±0	0.0008±0.0002
**Hybrid 9**	0.0068±0.0029	0.0171±0.0069	0.0635±0.0061	0.0646±0.0149	0.014±0.0198	0.0519±0.0223	0.0242±0.0045	0.0059±0.0019	0.0004±0.0001	0.0008±0.0004

Supplementary Table 2: Free Amino Acid Values (g/100g) in Raw Kernel Flour. Free amino acid values of all twenty amino acids are recorded. Standard deviations were calculated by two-six biological replications, dependent on genotype.

	Lys	Met	Phe	Pro	Ser	Trp	Thr	Tyr	Val	Cys
**Popcorn Parent 4**	0.0031±0.0005	0.0001±0	0.0006±0	0.0138±0.0009	0.0011±0.0001	0.0013±0	0.0008±0.0001	0.0024±0.0001	0.0011±0.0001	0.0001±0
**Popcorn Parent 3**	0.0038±0.0005	0.0002±0	0.002±0.0003	0.052±0.0046	0.0077±0.0004	0.0008±0	0.002±0	0.0032±0.0002	0.0015±0	0.0001±0
**Popcorn Parent 1**	0.0029±0.0001	0.0002±0.0001	0.0008±0.0001	0.0135±0.0073	0.0022±0.0008	0.001±0.0001	0.0009±0.0002	0.0021±0.0002	0.0011±0.0001	0.0001±0
**Popcorn Parent 2**	0.0028±0.0005	0.0001±0	0.0005±0.0001	0.0043±0.0031	0.0027±0.0012	0.0008±0.0001	0.0008±0.0002	0.0017±0.0002	0.0007±0	0.0001±0
**B73**	0.0029±0.0005	0.0004±0.0001	0.0011±0.0001	0.0393±0.0102	0.0021±0.0006	0.001±0.0002	0.0007±0.0002	0.0049±0.0024	0.0015±0.0001	0.0001±0
**CML154Q**	0.0266±0.0058	0.0029±0.0015	0.0094±0.0033	0.1691±0.0681	0.0151±0.009	0.0035±0.0009	0.0113±0.0051	0.0304±0.0082	0.0094±0.0032	0.0005±0
**K0326Y**	0.0214±0.01	0.0007±0.0007	0.0027±0.0019	0.0814±0.0286	0.0111±0.0081	0.0019±0.0003	0.0047±0.0031	0.0096±0.003	0.0045±0.0032	0.0002±0
**QPP Inbred 1**	0.0162±0.0071	0.0035±0.0032	0.0053±0.0034	0.1297±0.0423	0.0155±0.0108	0.0033±0.0024	0.0191±0.0156	0.0128±0.0063	0.0076±0.0041	0.0005±0.0003
**QPP Inbred 10**	0.006±0.0003	0.0001±0	0.0008±0	0.1015±0.0115	0.0009±0.0001	0.0025±0.0001	0.0018±0.0004	0.0047±0.0006	0.0017±0.0001	0.0001±0
**QPP Inbred 11**	0.0177±0.0028	0.0007±0.0005	0.0048±0.0006	0.1554±0.0323	0.0126±0.0046	0.0027±0.0011	0.0166±0.0017	0.0121±0.0017	0.0053±0.0022	0.0008±0.0001
**QPP Inbred 12**	0.0315±0.0079	0.0036±0.0007	0.0048±0	0.1205±0.0086	0.0335±0.006	0.002±0.0001	0.0317±0.0042	0.0092±0.0005	0.0116±0.0023	0.0011±0.0002
**QPP Inbred 2**	0.0123±0.0003	0.001±0.0008	0.0029±0.0009	0.1267±0.0177	0.0071±0.0023	0.0026±0.0006	0.0065±0.0002	0.0098±0.0029	0.0034±0.0005	0.0003±0
**QPP Inbred 3**	0.0226±0.0027	0.0016±0.0005	0.0055±0.0021	0.0986±0.0713	0.0238±0.0147	0.0043±0.0005	0.021±0.003	0.0174±0.0041	0.007±0.0027	0.0005±0.0001
**QPP Inbred 4**	0.0302±0.0037	0.0021±0.0006	0.0068±0.0001	0.1509±0.0013	0.0255±0.0052	0.0034±0.0005	0.0286±0.0059	0.0182±0.0017	0.0111±0.0015	0.0009±0.0001
**QPP Inbred 5**	0.0155±0.0064	0.0018±0.0012	0.0039±0.0016	0.1041±0.0349	0.0105±0.0043	0.0023±0.0003	0.0085±0.0032	0.0085±0.0024	0.0052±0.0022	0.0003±0.0001
**QPP Inbred 6**	0.0141±0.0055	0.0007±0.0003	0.0024±0.001	0.1116±0.0352	0.0058±0.0003	0.0027±0.0004	0.0049±0.0021	0.007±0.0015	0.0043±0.0022	0.0004±0.0002
**QPP Inbred 7**	0.0486±0.0072	0.0055±0.0007	0.0066±0.0011	0.112±0.0118	0.0384±0.0033	0.0044±0.0004	0.0344±0.0006	0.0217±0.0002	0.0156±0.0001	0.0004±0.0002
**QPP Inbred 8**	0.0257±0.0079	0.0008±0.0008	0.0022±0.0016	0.0809±0.0173	0.0052±0.0031	0.0032±0.0006	0.0059±0.0046	0.0101±0.004	0.0026±0.0017	0.0004±0.0003
**QPP Inbred 9**	0.0089±0.0019	0.0001±0	0.0011±0.0004	0.0853±0.0183	0.0014±0.0008	0.0017±0.0001	0.0021±0.0002	0.005±0.0004	0.0023±0	0.0001±0
**Tx807**	0.0254±0.0013	0.0006±0.0001	0.0054±0.0001	0.0385±0.006	0.0107±0.0011	0.0028±0.0002	0.0112±0.0015	0.0134±0.0014	0.005±0.0005	0.0004±0
**Hybrid 19**	0.0116±0.0025	0.0002±0	0.0019±0.0008	0.0717±0.0161	0.0028±0.0016	0.0027±0.0004	0.0035±0.0022	0.0065±0.0015	0.0019±0.0005	0.0001±0
**Hybrid 20**	0.0126±0.0016	0.0002±0	0.0021±0.0006	0.078±0.023	0.0028±0.0015	0.003±0.0004	0.0037±0.0015	0.0087±0.0024	0.0022±0.0003	0.0001±0
**Hybrid 23**	0.0115±0.004	0.0006±0.0004	0.0047±0.0023	0.1035±0.0246	0.0078±0.005	0.0029±0.0005	0.0096±0.0056	0.0122±0.0035	0.0041±0.0015	0.0003±0.0001
**Hybrid 25**	0.015±0.005	0.0007±0.0007	0.0049±0.0032	0.0957±0.0256	0.0096±0.0095	0.0035±0.0003	0.0094±0.0081	0.0166±0.0087	0.0051±0.0034	0.0003±0.0001
**Hybrid 28**	0.0123±0.0024	0.0002±0	0.0017±0.0011	0.0686±0.0188	0.0027±0.0016	0.0024±0.0001	0.0033±0.0023	0.0076±0.0029	0.0021±0.0006	0.0001±0
**Hybrid 30**	0.0085±0.0025	0.0002±0.0001	0.0023±0.0013	0.1095±0.0128	0.0038±0.0032	0.0024±0.0004	0.005±0.0039	0.0121±0.0049	0.0026±0.0014	0.0002±0
**Hybrid 38**	0.0137±0.0033	0.0001±0	0.0017±0.0011	0.0673±0.0102	0.0025±0.0015	0.0029±0.0005	0.0032±0.0015	0.0089±0.0033	0.0019±0.0003	0.0002±0
**Hybrid 43**	0.0153±0.0048	0.0003±0.0001	0.0034±0.0013	0.106±0.0285	0.0042±0.0023	0.0031±0.0003	0.0055±0.0036	0.0143±0.0037	0.003±0.0007	0.0002±0.0001
**Hybrid 8**	0.0096±0.0016	0.0001±0	0.0021±0.0014	0.0655±0.0176	0.0023±0.0014	0.0022±0.0005	0.0032±0.0016	0.006±0.0028	0.0015±0.0002	0.0002±0
**Hybrid 9**	0.0096±0.0036	0.0002±0.0001	0.0016±0.0008	0.0505±0.0172	0.0026±0.0015	0.0025±0.0003	0.0038±0.0022	0.0063±0.0021	0.002±0.0006	0.0002±0

Supplementary Table 2 Continued: Free Amino Acid Values (g/100g) in Raw Kernel Flour. Free amino acid values of all twenty amino acids are recorded. Standard deviations were calculated by two-six biological replications, dependent on genotype.

	Hybrid	Germination Rate	Days to Pollinating	Rot Susceptibility	Number of Ears Harvested	Ear Length	Number of Rows per Ear	Ear Weight	Kernel Size	Vitreousness Level	Hundred Grain Weight	Pop-Ability	Expansion Volume
	1	-0.037	2.076	-0.022	-1.142	-3.261	-1.841	-18.283	10.831	0.855	-1.686	0.016	94.932
	2	-0.270	2.976	0.024	-4.236	-1.642	-2.027	-25.041	16.319	0.395	-2.060	0.005	99.905
	3	0.140	-1.004	0.053	0.081	-1.501	-0.167	-5.694	2.639	-0.595	-0.528	-0.002	-8.857
	4	0.097	1.163	0.036	0.297	-1.666	-0.034	-3.716	3.886	-0.025	-0.727	0.002	-17.113
	5	-0.078	3.825	0.028	-2.221	-4.573	-2.370	-29.536	27.055	0.316	-2.879	0.017	112.069
	6	-0.510	1.422	-0.013	-5.603	-2.622	-1.195	-2.570	-1.981	-0.074	0.049	0.006	3.366
	7	0.104	2.304	-0.049	1.089	-2.909	-0.581	-14.350	16.520	-0.106	-2.071	0.010	52.473
	8	0.160	-1.797	-0.047	1.808	2.376	0.546	9.909	-2.967	0.903	0.270	0.010	28.098
	9	0.128	-0.472	0.019	0.801	2.345	0.621	8.096	-1.805	0.819	0.111	0.020	71.737
	10	0.202	-0.810	0.020	0.945	-1.237	0.223	-6.276	5.986	-0.908	-0.947	-0.003	-59.179
	11	0.197	0.175	0.067	1.089	-1.711	-0.997	-7.679	0.038	-0.166	-0.245	-0.059	-100.851
	12	0.114	2.418	0.022	0.153	-4.015	-2.575	-23.254	10.553	0.382	-1.495	0.006	85.890
	13	0.061	3.368	-0.010	0.657	-1.715	-1.782	-19.941	9.818	0.198	-1.264	0.015	53.259
	14	0.135	-0.852	0.078	0.585	-1.687	-0.293	-7.342	2.226	-1.077	-0.491	-0.004	-18.292
	15	0.165	0.517	0.063	1.376	-3.159	0.116	-9.107	9.699	-0.209	-1.124	-0.008	-10.429
	16	-0.027	3.140	-0.004	-1.142	-3.597	-2.476	-29.301	31.190	0.791	-3.200	0.022	119.700
	17	0.162	1.962	0.027	1.017	-2.324	-0.533	-8.613	-0.029	-0.077	-0.210	0.013	28.491
	18	0.129	2.342	0.013	0.009	-2.745	-0.925	-16.935	15.242	0.043	-1.838	0.021	74.095
	19	0.141	-2.677	0.035	2.168	2.127	-0.225	9.178	-6.284	0.668	0.716	0.015	35.934
	20	0.157	-2.487	0.028	1.520	2.637	0.204	6.771	-5.455	0.717	0.710	0.019	50.114
	21	0.046	-0.624	0.030	0.009	-1.884	-0.298	-6.388	1.070	-0.642	-0.267	-0.003	-29.693
	22	0.139	-0.244	0.120	0.585	-1.528	-0.645	-4.402	-2.503	0.044	0.265	-0.038	-56.820
	23	0.040	-1.042	0.002	1.664	0.943	-0.725	12.595	-10.644	-0.262	1.591	-0.032	-30.873
	24	-0.168	-0.092	-0.013	-0.782	1.795	-0.287	9.770	-6.910	-0.378	0.970	-0.013	-34.411
	25	0.051	-1.156	-0.075	1.880	1.637	1.413	16.628	-7.374	-0.191	0.937	-0.001	-39.129
	26	0.029	0.479	-0.055	0.081	1.068	1.838	17.377	-8.543	-0.436	1.094	-0.010	-54.068
	27	-0.138	-1.427	-0.101	-0.830	2.318	-0.661	7.919	-8.657	0.646	1.153	-0.003	-8.464
	28	0.030	-2.826	0.072	1.376	2.702	-0.586	9.367	-6.365	0.929	0.668	0.014	48.935
	29	-0.037	-0.320	-0.023	-0.494	3.106	0.492	16.712	-15.752	-0.652	2.548	-0.012	-76.084
	30	0.055	0.897	-0.001	0.729	2.061	-0.362	4.791	-2.810	-0.246	0.176	-0.009	2.151
	31	-0.205	-0.814	-0.075	-1.286	1.223	0.849	-6.119	4.893	0.091	-0.657	0.018	-14.754
	32	-0.006	-0.510	-0.079	0.801	1.141	1.718	18.423	-9.479	-0.355	1.244	-0.014	-69.400
	33	-0.054	-0.890	-0.113	0.873	3.313	1.427	18.797	-9.928	0.213	1.740	-0.046	-109.500
	34	0.093	-2.753	0.034	1.520	1.175	0.204	11.252	-11.778	-1.359	1.912	-0.017	-67.435
	35	-0.153	0.061	0.016	-0.998	0.505	0.920	8.333	-5.632	-1.206	0.859	-0.028	-76.084
	36	-0.268	-0.966	-0.025	-1.646	1.139	1.787	7.619	-4.281	-0.635	0.503	0.014	-24.976
	37	-0.247	0.669	0.017	-1.430	-0.497	1.475	2.915	-2.226	-0.224	0.206	0.003	-21.830
	38	0.144	-2.411	0.065	1.448	1.603	0.151	5.027	-4.594	1.098	0.312	0.023	57.977
	39	-0.121	-0.481	-0.020	-0.782	1.752	0.140	1.746	-2.834	0.956	0.154	0.022	68.198
	40	-0.185	0.061	-0.036	-1.933	1.563	1.101	4.903	-9.084	-0.349	1.252	0.012	-26.155
	41	-0.079	-1.194	-0.017	-0.351	1.420	0.693	5.212	-4.896	-0.545	0.426	-0.009	-15.933
	42	-0.169	0.061	-0.039	-1.646	0.571	1.405	-0.890	-1.759	0.310	0.150	0.002	-26.155
	43	0.084	-1.139	-0.017	1.592	1.000	2.265	11.506	-3.534	0.006	0.360	-0.006	-56.820
	44	-0.048	-0.928	-0.036	0.369	2.752	1.998	20.589	-9.862	0.340	1.316	0.013	-34.018
**Standard Error**		0.006	0.772	0.002	0.731	1.17	0.366	41.73	23.967	0.0874	4.444	0.0001	862.378
**Genetic Repeatability**		0.566	0.465	0.078	0.345	0.716	0.673	0.728	0.676	0.684	0.683	0.201	0.582

Supplementary Table 3: Specific Combining Ability (SCA) and genetic repeatability estimates for all recorded traits. Specific Combining Ability and genetic repeatability estimates were found with ASReml-R software. High SCAs were noted in elite hybrids, shaded in gray. High repeatabilities were calculated for ear length and ear weight.

	Ala	Arg	Asx	Glx	Gly	His	Ile	Leu	Lys	Met	Phe	Pro	Ser	Thr	Tyr	Val
**Popcorn Parent 4**	1.254± 0.144	0.365± 0.061	0.807± 0.057	3.085± 0.087	1.159± 0.076	0.451± 0.022	0.783± 0.073	2.48± 0.194	0.206± 0.043	0.193± 0.029	0.809 ±0.033	1.291± 0.091	0.773± 0.074	0.6± 0.071	0.486 ±0.053	0.66± 0.044
**Popcorn Parent 3**	1.056± 0.024	0.31± 0.024	0.7± 0.031	2.591± 0.092	0.909± 0.018	0.402± 0.009	0.659± 0.029	2.006± 0.117	0.173± 0.017	0.196± 0.005	0.698± 0.018	1.168± 0.06	0.667± 0.023	0.547± 0.034	0.445± 0.031	0.555± 0.018
**Popcorn Parent 1**	1.248± 0.037	0.379± 0.022	0.795± 0.026	3.077± 0.165	1.089± 0.016	0.499± 0.017	0.755± 0.053	2.388± 0.148	0.197± 0.013	0.259± 0.035	0.788± 0.025	1.386± 0.054	0.783± 0.048	0.616± 0.027	0.515± 0.043	0.65± 0.026
**Popcorn Parent 2**	0.991± 0.1	0.295± 0.021	0.662± 0.04	2.58± 0.139	1.003± 0.107	0.391± 0.018	0.642± 0.038	1.978± 0.152	0.201± 0.028	0.182± 0.015	0.707± 0.035	1.146± 0.052	0.642± 0.047	0.484± 0.054	0.425± 0.024	0.527± 0.029
**QPP Hybrid 20**	0.752± 0.052	0.514± 0.038	0.852± 0.122	2.234± 0.188	0.862± 0.139	0.552± 0.024	0.54± 0.038	1.342± 0.099	0.402± 0.024	0.128± 0.006	0.603± 0.04	1.128± 0.05	0.59± 0.032	0.527± 0.029	0.322± 0.037	0.628± 0.036
**QPP Hybrid 25**	0.709± 0.066	0.494± 0.078	0.973± 0.219	2.24± 0.171	0.936± 0.127	0.553± 0.054	0.501± 0.047	1.164± 0.086	0.37± 0.065	0.135± 0.023	0.532± 0.043	1.113± 0.079	0.556± 0.066	0.508± 0.039	0.294± 0.045	0.609± 0.061
**QPP Hybrid 28**	0.872± 0.079	0.558± 0.072	0.829± 0.119	2.57± 0.229	0.935± 0.151	0.614± 0.027	0.622± 0.051	1.581± 0.164	0.358± 0.061	0.137± 0.012	0.672± 0.069	1.297±0.0 67	0.653± 0.05	0.571± 0.041	0.39± 0.061	0.708± 0.048
**QPP Hybrid 38**	0.713± 0.03	0.553± 0.057	0.778± 0.11	2.189± 0.103	1.282± 0.137	0.644± 0.05	0.517± 0.025	1.359± 0.058	0.371± 0.04	0.117± 0.011	0.555± 0.029	1.26± 0.049	0.554± 0.04	0.519± 0.031	0.338± 0.025	0.617± 0.035
**QPP Hybrid 43**	0.758± 0.087	0.63± 0.155	0.915± 0.248	2.348± 0.32	1.292± 0.096	0.641± 0.094	0.546± 0.086	1.326± 0.175	0.42± 0.077	0.148± 0.025	0.559± 0.058	1.269± 0.121	0.599± 0.099	0.571± 0.068	0.357± 0.044	0.651± 0.097

Supplementary Table 4: Protein-Bound Amino Acid Levels (g/100g) in Air Popped Flakes. Protein-bound amino acid values of sixteen amino acids in air popped flakes are recorded. Aspartate and asparagine (Asx), glutamine and glutamate (Glx), Serine, and Tryptophan are destroyed during acidic hydrolysis, the procedure used for amino acid quantification. Only five QPP hybrids and four popcorn parents were tested with air popping. Standard deviations were calculated by four biological replications.

	Ala	Arg	Asx	Glx	Gly	His	Ile	Leu	Lys	Met	Phe	Pro	Ser	Thr	Tyr	Val
**Popcorn Parent 1**	1.314±0.085	0.336±0.039	0.838±0.091	3.185±0.264	1.101±0.122	0.502±0.04	0.823±0.091	2.555±0.285	0.176±0.024	0.274±0.025	0.821±0.071	1.431±0.115	0.776±0.059	0.612±0.068	0.537±0.08	0.679±0.066
**Popcorn Parent 2**	0.967±0.054	0.249±0.031	0.614±0.073	2.485±0.163	0.843±0.092	0.38±0.027	0.603±0.062	1.917±0.123	0.155±0.014	0.171±0.01	0.668±0.047	1.098±0.056	0.593±0.058	0.451±0.041	0.374±0.043	0.506±0.044
**QPP Hybrid 20**	0.849±0.052	0.504±0.073	0.872±0.079	2.479±0.154	0.911±0.14	0.582±0.042	0.608±0.063	1.517±0.165	0.357±0.078	0.148±0.025	0.662±0.064	1.22±0.087	0.632±0.06	0.565±0.059	0.353±0.042	0.682±0.063
**QPP Hybrid 25**	0.748±0.044	0.466±0.041	0.831±0.084	2.325±0.056	0.982±0.127	0.568±0.021	0.524±0.033	1.255±0.045	0.313±0.011	0.138±0.016	0.569±0.017	1.138±0.057	0.558±0.035	0.524±0.025	0.31±0.022	0.64±0.035
**QPP Hybrid 28**	0.715±0.075	0.506±0.062	0.688±0.119	2.195±0.168	1.196±0.017	0.658±0.031	0.511±0.06	1.345±0.121	0.287±0.038	0.117±0.012	0.527±0.063	1.2±0.034	0.537±0.059	0.502±0.051	0.32±0.056	0.596±0.056
**QPP Hybrid 38**	0.695±0.054	0.558±0.059	0.788±0.108	2.099±0.136	1.197±0.034	0.593±0.061	0.503±0.028	1.268±0.115	0.334±0.031	0.118±0.012	0.53±0.033	1.163±0.096	0.545±0.044	0.509±0.034	0.317±0.034	0.591±0.034
**QPP Hybrid 43**	0.726±0.077	0.577±0.024	0.869±0.114	2.276±0.222	1.253±0.054	0.627±0.06	0.527±0.046	1.301±0.139	0.353±0.074	0.139±0.022	0.538±0.05	1.216±0.078	0.548±0.049	0.532±0.019	0.331±0.045	0.622±0.062

Supplementary Table 5: Protein-Bound Amino Acid Levels (g/100g) in Microwaved Popped Flakes. Protein-bound amino acid values of sixteen amino acids in microwave-popped flakes are recorded. Aspartate and asparagine (Asx), glutamine and glutamate (Glx), Serine, and Tryptophan are destroyed during acidic hydrolysis, the procedure used for amino acid quantification. Only five QPP hybrids and two popcorn parents were tested with air popping. Standard deviations were calculated by four biological replications.

	Ala	Arg	Asx	Glx	Gly	His	Ile	Leu	Lys	Met	Phe	Pro	Ser	Thr	Tyr	Val
**Popcorn Parent 1**	0.897±0.195	0.267±0.021	0.601±0.083	2.269±0.389	0.773±0.172	0.364±0.044	0.529±0.114	1.751±0.358	0.188±0.022	0.179±0.046	0.625±0.102	1.067±0.176	0.575±0.101	0.459±0.077	0.38±0.089	0.48±0.076
**Popcorn Parent 2**	0.855±0.146	0.296±0.073	0.635±0.158	2.187±0.422	0.745±0.187	0.351±0.076	0.548±0.136	1.662±0.338	0.23±0.082	0.152±0.033	0.63±0.116	0.969±0.17	0.571±0.115	0.454±0.099	0.354±0.108	0.503±0.116
**QPP Hybrid 20**	0.611±0.078	0.379±0.025	0.672±0.101	1.8±0.24	0.77±0.08	0.474±0.05	0.428±0.046	1.076±0.154	0.266±0.018	0.098±0.007	0.487±0.06	0.945±0.103	0.475±0.064	0.414±0.035	0.269±0.018	0.519±0.05
**QPP Hybrid 25**	0.543±0.051	0.363±0.097	0.686±0.12	1.78±0.192	0.645±0.098	0.481±0.059	0.391±0.047	0.927±0.078	0.28±0.05	0.097±0.01	0.425±0.049	0.894±0.089	0.426±0.06	0.398±0.037	0.239±0.038	0.503±0.058
**QPP Hybrid 28**	0.565±0.105	0.485±0.135	0.586±0.088	1.675±0.224	1.014±0.151	0.499±0.036	0.396±0.058	0.989±0.151	0.273±0.027	0.088±0.014	0.434±0.062	0.935±0.083	0.433±0.053	0.399±0.027	0.245±0.039	0.488±0.053
**QPP Hybrid 38**	0.62±0.088	0.487±0.013	0.677±0.055	1.807±0.324	1.117±0.107	0.529±0.098	0.437±0.057	1.123±0.238	0.264±0.016	0.102±0.012	0.481±0.056	0.983±0.225	0.496±0.05	0.448±0.041	0.29±0.067	0.529±0.054
**QPP Hybrid 43**	0.662±0.023	0.567±0.061	0.823±0.191	2.007±0.155	1.141±0.076	0.589±0.048	0.466±0.021	1.152±0.059	0.345±0.077	0.117±0.006	0.493±0.022	1.089±0.079	0.509±0.042	0.495±0.048	0.26±0.016	0.561±0.034

Supplementary Table 6: Protein-Bound Amino Acid Levels (g/100g) in Oil Popped Flakes. Protein-bound amino acid values of sixteen amino acids in oil-popped flakes are recorded. Aspartate and asparagine (Asx), glutamine and glutamate (Glx), serine, and tryptophan are destroyed during acidic hydrolysis, the procedure used for protein-bound amino acid quantification. Only five QPP hybrids and two popcorn parents were tested with air popping. Standard deviations were calculated by four biological replications.

	Ala	Arg	Asn	Asp	Gln	Glu	Gly	His	Ile	Leu	Lys	Met	Phe	Pro	Ser	Trp	Thr	Tyr	Val	Cys
**Popcorn Parent 4**	0.000806±0.00028	0.00121±0.000196	0.00133±0.00111	0.000792±0.0003	0±0	0.000194±0.00010	0.000823±0.00041	0.000599±0.00015	0.0000868±0.00000173	0.0000288±0.0000498	0.000642±0.00024	0±0	0.000109±0.00000218	0.00137±0.000914	0.000278±0.0000662	0.000135±0.00000269	0.000211±0.0000961	0.0000791±0.0000685	0.0000257±0.0000445	0.0000539±0.0000467
**Popcorn Parent 3**	0.00119±0.0000386	0.00128±0.000163	0.0014±0.000284	0.00331±0.000461	0±0	0.000795±0.000151	0.00196±0.0019	0.00113±0.0000851	0.000085±0.000000498	0.000114±0.00005	0.00115±0.000369	0±0	0.00025±0.0000634	0.00996±0.00355	0.000705±0.000162	0.000132±0.000000775	0.00155±0.000955	0.000313±0.000069	0.0000759±0.000000445	0.0000522±0.0000452
**Popcorn Parent 1**	0.000764±0.00022	0.00149±0.00017	0.00472±0.000973	0.0022±0.00037	0±0	0.00067±0.000207	0.00146±0.000794	0.000742±0.000133	0.0000666±0.0000444	0.0000667±0.0000445	0.000738±0.0000911	0.0000247±0.0000495	0.000111±0.00000172	0.00258±0.000852	0.000337±0.000107	0.000138±0.00000213	0.000141±0.0000413	0.000122±0.00000189	0.0000596±0.0000397	0.0000817±0.00000126
**Popcorn Parent 2**	0.000512±0.00012	0.00132±0.000171	0.00365±0.00201	0.00199±0.000666	0±0	0.0012±0.000591	0.00115±0.00059	0.000657±0.00021	0.0000647±0.00004	0.0000864±0.00000123	0.000753±0.00021	0±0	0.000109±0.00000155	0.00192±0.00114	0.000519±0.00012	0.000168±0.0000646	0.000137±0.0000381	0.000239±0.0000034	0.0000575±0.0000384	0.0000798±0.00000114
**QPP Hybrid 20**	0.00235±0.000557	0.00993±0.00437	0.0277±0.00573	0.0305±0.0109	0±0	0.0076±0.00541	0.00361±0.00244	0.00206±0.000355	0.00011±0.0000469	0.000152±0.0000794	0.00314±0.000935	0.0000242±0.0000484	0.000518±0.00038	0.0296±0.0126	0.000697±0.00034	0.00068±0.000239	0.00645±0.00226	0.00201±0.000953	0.000487±0.000122	0.000221±0.0000729
**QPP Hybrid 25**	0.00536±0.00225	0.00833±0.00149	0.0337±0.00797	0.0581±0.013	0.0000712±0.00014	0.015±0.00615	0.00647±0.00183	0.00318±0.000803	0.000321±0.00023	0.0015±0.00132	0.00457±0.00112	0.000244±0.00017	0.00245±0.00147	0.0421±0.0159	0.00458±0.0044	0.00133±0.000274	0.0103±0.00426	0.00936±0.00542	0.0021±0.00165	0.000237±0.00011
**QPP Hybrid 28**	0.00177±0.000798	0.00675±0.00361	0.0197±0.00976	0.0265±0.0174	0±0	0.00412±0.00562	0.00207±0.00263	0.00186±0.000655	0.0000427±0.00005	0.000129±0.0000827	0.00247±0.000967	0±0	0.000348±0.00041	0.0199±0.0161	0.000636±0.00043	0.000433±0.00042	0.00411±0.00427	0.00148±0.00159	0.000287±0.00026	0.000198±0.00015
**QPP Hybrid 38**	0.00234±0.000545	0.00481±0.0026	0.0244±0.00603	0.034±0.0107	0±0	0.00415±0.00179	0.00256±0.00184	0.00176±0.000496	0.0000867±0.00000114	0.000109±0.0000439	0.00271±0.000944	0±0	0.000465±0.00029	0.0267±0.0123	0.000607±0.00023	0.000641±0.00017	0.00688±0.0029	0.00165±0.000541	0.000465±0.00011	0.000279±0.00015
**QPP Hybrid 43**	0.00402±0.0017	0.00738±0.00441	0.0259±0.0146	0.0376±0.0218	0.0000735±0.0000945	0.011±0.0105	0.00467±0.00392	0.00234±0.000952	0.000198±0.00017	0.000441±0.00060	0.00376±0.00228	0.000125±0.00019	0.00127±0.00116	0.0525±0.0181	0.00178±0.00193	0.000817±0.00040	0.0137±0.00617	0.00432±0.00275	0.00104±0.00109	0.000649±0.00044

Supplementary Table 7: Free Amino Acid Levels (g/100g) in Air Popped Flakes. Free amino acid values of all twenty amino acids are recorded. As shown, all free amino acids substantially decline in abundance after popping. The five elite QPP hybrids and four popcorn parents were popped by air. Standard deviations were calculated by four biological replications of each genotype.

	Ala	Arg	Asn	Asp	Gln	Glu	Gly	His	Ile	Leu	Lys	Met	Phe	Pro	Ser	Trp	Thr	Tyr	Val	Cys
**Popcorn Parent 1**	0.0009482463±0.0002365559	0.001741848±0.0004865403	0.0052037587±0.0020342478	0.0022507094±0.0007099502	0±0	0.0003721632±0.0001004849	0.0018393288±0.0008881289	0.0007413003±0.0001857273	0.00002249914±0.00004499828	0.00002246062±0.00004492123	0.0008330781±0.0002889226	0.0000242224±0.00004844481	0.0001111534±0.000002824104	0.0037565672±0.001144277	0.0002834253±0.00006064005	0.0001042685±0.00006952621	0.000201172±0.00008390191	0.00009250559±0.00006168268	0.00005981031±0.00003988149	0.00008152643±0.000002071363
**Popcorn Parent 2**	0.000431431±0.0001754707	0.00119251±0.0002272991	0.0009401704±0.0002164053	0.0007320231±0.0001078965	0±0	0.0001904881±0.000002713075	0.0005427041±0.0006295066	0.0005418658±0.0001880436	0±0	0.00006385784±0.00004258655	0.000531634±0.0001499558	0±0	0.0001069351±0.000001523051	0.0006483133±0.0005851235	0.0002383546±0.00004142166	0.0001322075±0.000001882999	0.00007711189±0.00000109829	0.00008820921±0.00005882639	0±0	0.00007843248±0.00000111709
**QPP Hybrid 20**	0.0026273338±0.0005253412	0.005271028±0.0009995258	0.0236457545±0.0043510332	0.0333104174±0.0066603816	0±0	0.002416842±0.00130196	0.0022047871±0.0015269503	0.0019492611±0.0001685865	0.0001074379±0.00004478807	0.0002569593±0.00009933126	0.0023867759±0.0002357523	0±0	0.0006469428±0.0002638239	0.0241894477±0.007442605	0.0007720446±0.0002609134	0.0005334992±0.0001889074	0.004755781±0.001710336	0.002779387±0.0006441274	0.0004977861±0.0001606601	0.0001584233±0.00006633965
**QPP Hybrid 25**	0.0045106601±0.0024204423	0.006151139±0.0019706678	0.0261335466±0.0071172068	0.0394566982±0.0137240549	0.00004753136±0.0000548916	0.0028958854±0.001870876	0.0029132709±0.001264521	0.0023100025±0.0007745874	0.0001692952±0.0002281432	0.0008056195±0.000959592	0.0029598845±0.0009597532	0.0001446011±0.0001659447	0.0014719689±0.001014932	0.024289897±0.0042562222	0.0028388085±0.003157153	0.0008609623±0.0003166468	0.004945829±0.001569736	0.005635869±0.003678759	0.001099631±0.001051773	0.0001375024±0.00003958659
**QPP Hybrid 28**	0.0022881962±0.0004643949	0.003146165±0.0009619635	0.0175810897±0.004551998	0.0250493416±0.0076961613	0±0	0.001811986±0.000698582	0.0027110223±0.0028351934	0.0016151557±0.0004456378	0.00006571855±0.00004389156	0.0001755595±0.0001256886	0.0022014835±0.0007000656	0.00002528983±0.00005057966	0.0005790401±0.0004761225	0.0138389265±0.0048636911	0.0008664472±0.0008493801	0.0004778727±0.0001751826	0.003173539±0.001545485	0.00171929±0.001118404	0.0004122344±0.0002708196	0.0001418729±0.00004030057
**QPP Hybrid 38**	0.0020741728±0.0007131781	0.003509522±0.0014794624	0.0231666703±0.0081097997	0.0265255607±0.0124497166	0±0	0.0021857841±0.001273317	0.0013758317±0.0010168604	0.0015060134±0.0004030024	0.00008564974±0.000000160956	0.000107494±0.00008178895	0.0021156913±0.0005635579	0±0	0.0004596332±0.0002973852	0.0155259675±0.0060488209	0.0005322905±0.0003029079	0.000468173±0.000138271	0.003656642±0.001646911	0.001514608±0.0009443404	0.000249589±0.0001314453	0.0001388724±0.00004101482
**QPP Hybrid 43**	0.0054818223±0.003260012	0.006316167±0.0027925839	0.026418924±0.0116961777	0.0374209989±0.0220457084	0.0002341987±0.0004683974	0.0086145505±0.01231544	0.003111714±0.0032355611	0.0026129592±0.0014820626	0.0001697118±0.0001180368	0.0004661569±0.0004436924	0.0039654384±0.0021068802	0.0001680829±0.0002261091	0.001335426±0.0009928845	0.0406693458±0.0252109382	0.0021879253±0.00217686	0.0009253966±0.0005606712	0.009339466±0.006971784	0.004602706±0.003060331	0.001095781±0.001152142	0.00003911748±0.00004517136

Supplementary Table 8: Free Amino Acid Levels (g/100g) in Microwave Popped Flakes. Free amino acid values of all twenty amino acids in microwave-popped flakes are recorded. As shown, all free amino acids substantially decline in abundance after popping. The five elite hybrids and two popcorn parents were popped with microwave and oil popping methods. Standard deviations were calculated by four biological replications of each genotype.

	Ala	Arg	Asn	Asp	Gln	Glu	Gly	His	Ile	Leu	Lys	Met	Phe	Pro	Ser	Trp	Thr	Tyr	Val	Cys
**Popcorn Parent 1**	0.0006055715±0.000089926	0.001361282±0.000379367	0.007555273±0.001326479	0.00205416±0.0004704377	0±0	0.0008027696±0.0001185045	0.001070521±0.0008517239	0.0006586571±0.0001731321	0±0	0.00006513198±0.0000434933	0.0007262074±0.0001781827	0.000195922±0.0001364582	0.0001093286±0.00000315079	0.0032155498±0.0009254667	0.000260839±0.00003583853	0.0001351667±0.00000389543	0.0001193118±0.0000796212	0.000119918±0.000003455971	0.00007753404±0.0000022345	0.000080188±0.000002310975
**Popcorn Parent 2**	0.000478017±0.0002365074	0.001503335±0.0002000403	0.004152039±0.002450013	0.001035401±0.0004524599	0±0	0.0007145323±0.0003522831	0.001205814±0.0003073448	0.000678501±0.0001855328	0.00002136319±0.0000427264	0.00006330407±0.0000422092	0.0006798674±0.0001704428	0±0	0.0001071172±0.00000188884	0.0009911009±0.001300758	0.0004248041±0.0002161146	0.00009856362±0.0000657193	0.0001731916±0.00007290276	0.0001164347±0.00009468833	0.0000378238±0.00004367861	0.0000588331±0.00003924577
**QPP Hybrid 20**	0.0017704579±0.000349768	0.005062779±0.0028149051	0.019782365±0.007890235	0.027353054±0.0097435045	0±0	0.0043415193±0.0026988147	0.002749415±0.0012870895	0.0015724698±0.0003871151	0.00006629293±0.0000442062	0.0002200694±0.00009025278	0.0017294353±0.0006078962	0.00002537585±0.0000507517	0.0006376837±0.0002970466	0.0137838543±0.0057840178	0.0006703452±0.0003229067	0.0005126073±0.0002085285	0.0027059333±0.001501073	0.0019717565±0.0007426972	0.0003926669±0.0001716898	0.0001214359±0.00004748471
**QPP Hybrid 25**	0.0042690561±0.000699833	0.00579314±0.0009802109	0.027897233±0.004986444	0.043036181±0.011380583	0.0001874414±0.0002641226	0.0093543635±0.002863602	0.004923967±0.0020015564	0.0023030685±0.0004049921	0.0002992667±0.000141909	0.001337967±0.001049088	0.003382371±0.0005977175	0.0001440558±0.0001663501	0.0019076603±0.001067472	0.0333032687±0.0113730244	0.0032996147±0.002578605	0.001141168±0.0002281071	0.0074640906±0.001153801	0.0062570388±0.002253185	0.00133398±0.000698569	0.0001618139±0.000119189
**QPP Hybrid 28**	0.0016880979±0.000271718	0.004297051±0.0006235101	0.018520159±0.003325288	0.023771548±0.0121309901	0±0	0.003377691±0.0020479588	0.001755729±0.0017553255	0.0014256385±0.0003213544	0.0001063339±0.00004304956	0.0001698036±0.00006898908	0.001980856±0.0004819294	0±0	0.0004548921±0.0003536305	0.0207593688±0.0083449688	0.0005275476±0.0002563255	0.0004295147±0.0001246762	0.0034245118±0.002788864	0.0018163766±0.00134109	0.000398246±0.00009454382	0.00009780602±0.0000747207
**QPP Hybrid 38**	0.0015786804±0.000485819	0.003119074±0.0011731056	0.022855992±0.008387709	0.025123042±0.012998166	0±0	0.0047721435±0.0033670578	0.003007902±0.0017312362	0.0015691532±0.0005538586	0.00006402349±0.0000426869	0.000127875±0.0001095665	0.0021699272±0.0007644188	0.00002426179±0.0000485236	0.0005911391±0.0004997597	0.0148574978±0.0058599485	0.0006148385±0.0004543317	0.000498746±0.0002962318	0.0033889995±0.001750523	0.0016209766±0.001019124	0.0003803437±0.0002540361	0.0001575181±0.00006357525
**QPP Hybrid 43**	0.0031492882±0.001029297	0.007864514±0.0051672403	0.026885291±0.011574059	0.030405005±0.0152503882	0.00002481154±0.0000496231	0.0067119031±0.0063318403	0.003795633±0.0026524559	0.0018990164±0.0008719656	0.0001101307±0.00008499491	0.0002207845±0.0002247013	0.0036252118±0.0025363583	0.00002533277±0.0000506655	0.0008606107±0.0007323513	0.0378674777±0.0262502169	0.000956196±0.001145673	0.0006847551±0.00034193	0.0090652053±0.006734026	0.0030706223±0.002085509	0.0006703602±0.0005682421	0.0004283841±0.0003642795

Supplementary Table 9: Free Amino Acid Levels (g/100g) in Oil Popped Flakes. Free amino acid values of all twenty amino acids in oil-popped flakes are recorded. As shown, all free amino acids substantially decline in abundance after popping. The five elite hybrids and two popcorn parents were popped with microwave and oil popping methods. Standard deviations were calculated by four biological replications of each genotype.
